# A Multi-User Game-Theoretical Multipath Routing Protocol to Send Video-Warning Messages over Mobile *Ad Hoc* Networks

**DOI:** 10.3390/s150409039

**Published:** 2015-04-17

**Authors:** Ahmad Mohamad Mezher, Mónica Aguilar Igartua, Luis J. de la Cruz Llopis, Esteve Pallarès Segarra, Carolina Tripp-Barba, Luis Urquiza-Aguiar, Jordi Forné, Emilio Sanvicente Gargallo

**Affiliations:** 1 Department of Network Engineering, Universitat Politècnica de Catalunya (UPC),C. Jordi Girona 1-3, Barcelona 08034, Spain; E-Mails: monica.aguilar@entel.upc.edu (M.A.I.); luis.delacruz@entel.upc.edu (L.J.C.L.); esteve@entel.upc.edu (E.P.S.); luis.urquiza@entel.upc.edu (L.U.-A.); jforne@entel.upc.edu (J.F.); e.sanvicente@gmail.com (E.S.G.); 2 Faculty of Computer Science, Autonomous University of Sinaloa, Mazatlan 82107, Mexico; E-Mail: ctripp@uas.edu.mx

**Keywords:** Mobile ad hoc networks, Vehicular ad hoc networks, smart cities, adaptive multipath routing, game theory, Nash equilibrium, video-streaming services

## Abstract

The prevention of accidents is one of the most important goals of ad hoc networks in smart cities. When an accident happens, dynamic sensors (e.g., citizens with smart phones or tablets, smart vehicles and buses, *etc.*) could shoot a video clip of the accident and send it through the ad hoc network. With a video message, the level of seriousness of the accident could be much better evaluated by the authorities (e.g., health care units, police and ambulance drivers) rather than with just a simple text message. Besides, other citizens would be rapidly aware of the incident. In this way, smart dynamic sensors could participate in reporting a situation in the city using the ad hoc network so it would be possible to have a quick reaction warning citizens and emergency units. The deployment of an efficient routing protocol to manage video-warning messages in mobile Ad hoc Networks (MANETs) has important benefits by allowing a fast warning of the incident, which potentially can save lives. To contribute with this goal, we propose a multipath routing protocol to provide video-warning messages in MANETs using a novel game-theoretical approach. As a base for our work, we start from our previous work, where a 2-players game-theoretical routing protocol was proposed to provide video-streaming services over MANETs. In this article, we further generalize the analysis made for a general number of *N* players in the MANET. Simulations have been carried out to show the benefits of our proposal, taking into account the mobility of the nodes and the presence of interfering traffic.Finally, we also have tested our approach in a vehicular ad hoc network as an incipient start point to develop a novel proposal specifically designed for VANETs.

## 1. Introduction

A Mobile Ad hoc NETwok (MANET) is a group of self-organized wireless mobile nodes (MNs) able to communicate with each other without the need of any fixed network infrastructure nor centralized administrative support. MANETs suffer from link breakages due to nodes that move and have limited battery life, which produce frequent changes in the network topology. In addition, the transmission range in such mobile devices is limited, so multi-hop paths as well as efficient routing protocols will be needed. Each MANET node will operate both as a terminal host and as a forwarding node. MANETs should adapt dynamically to be able to maintain communications despite of all these issues [1].

MANETs have attracted much attention from the research community over the last years and important technical advances have risen. These multi-hop networks are foreseen as an important kind of next generation access networks, where multimedia services will be demanded by end users from their wireless devices everywhere. In many situations and areas, users may spontaneously form an infrastructureless ad hoc network to share their resources and contents. Besides, MANETs can be used together with existing cellular networks forming a hybrid cellular-ad hoc network as MANETs can extend the coverage, capacity and interconnectivity of current cellular networks [2]. Multimedia services require Quality of Service (QoS) provision. The special characteristics of MANETs, such as mobility, dynamic network topology, energy constraints, infrastructureless and variable link capacity, make the QoS provision over these networks an important challenge. In particular, instead of using fixed network configuration parameters, a better solution would be to adjust the framework parameters according to the current environmental conditions.

On the other hand, smart city applications are promising new services that involve vehicles, drivers, citizens and city infrastructure are about to emerge. Smart city applications could be used in emergency situations that require the coordination and collaboration between implicated parts in order to treat urgently the wounded who have suffered an accident. Emergency prevention and response are key issues for smart cities to face any kind of sudden incident such as traffic accidents, traffic jams, public transport delays, *etc*. For example, when an accident happens, dynamic sensors (e.g., citizens with smart phones) could make a video of the accident and send it through the MANET. With a video message, the level of seriousness of the accident could be better interpreted by the authorities (*i.e.*, health care unit, police, ambulance drivers, *etc.*) than with a simple text message. In this way, dynamic sensors could participate in reporting a situation in the city using the MANET network so it would be possible to have a quick answer warning citizens and emergency units. The prevention of accidents is one of the most important goals in smart cities, and nowadays Information and Communication Technologies (ICTs) and citizens play an essential role in this field.

A good management of video-warning messages after accidents would lead to an immediate call to health agencies and ambulances. In these kinds of situations, MANETs [1] and citizens play an important role in the new smart cities. Nowadays, a high percentage of citizens carry at least one smart communications device in their pockets or bags. In the smart cities, citizens are welcome to participate and interact with the management of their city. Diverse kind of data could be gathered from the smart phones or tablets of volunteers, e.g., mobility information or need for health care after an incident in the city. This way, the public administration would have an interactive network of citizens who actively participate in the management of the city. It would be possible to have a quick answer to solve daily problems and help the emergency units (e.g., police, ambulances, health care units) in case of incidents (e.g., traffic accidents).

Our research focuses on the deployment of an efficient multipath routing protocol to manage video-warning messages in MANETs. In this work, we aimed to design a dynamic selection of the data forwarding paths using a game-theoretical approach plus a multipath multimedia routing protocol that we previously introduced in [3]. This contribution seeks to further enhance the overall performance of the service.

The rest of the paper is structured as follows. Section 2 includes some relevant related work. In Section 3 we summarize the features of our multipath routing protocol. Section 4 gives a brief explanation of the game-theoretical proposal. Section 5 describes analytically our novel game-theoretical model to send video-warning messages over MANETs. Section 6 describes a method to calculate a specific parameter of the model. Simulation results are shown and analyzed in Section 7. Finally, conclusion and future work are given in Section 8.

## 2. Related Work

Routing is the process of selecting the best path or paths in a network through which data will be forwarded. Forwarding paths are not necessarily the shortest ones and are selected usually seeking to improve the service performance. Proposals of routing protocols for ad hoc networks similar to our work can be classified in four categories: (a) single path routing; (b) general multipath routing; (c) multipath routing used to transmit video; and (d) game-theoretical algorithms used in the routing. In the following we summarize some representative works related to our proposal for each category.

(a) Regarding single path routing, many protocols were designed in the last years for MANETs [4-10] based on improvements of basic and widely referenced ad hoc routing protocols such as ad hoc on-demand distance vector (AODV) [11] and dynamic source routing (DSR) [12]. Based on AODV, [4] incorporates an admission control scheme and a feedback scheme to satisfy the QoS-requirements of real-time applications; [5] adds a route fragility coefficient (RFC) as a metric to find stable routes; [6] proposes an efficient algorithm to balance energy consumption among all participating nodes; and [7] provides *n* backup routes in case of link failure instead of one. On the other side, based on DSR, [8] presents a pragmatic scheme to establish and sustain trustworthy routes in the network; [9] introduces a new route maintenance strategy called distance based route maintenance (DISTANCE) to prevent link failure; and [10] uses the ant colony optimization (ACO) algorithm producing a significantly improvement in terms of packet losses, end-to-end delay, routing overhead and energy consumption.
(b) Some works like [13–17] tackle the congestion problem present when the MANET is heavily loaded, since this factor negatively affects the packet losses. To cope with this issue, multipath routing protocols were proposed to alleviate congestion, optimize the use of the scarce MANET resources, increase the packet delivery ratio and improve the offered quality of service. For example, authors in [13] proposed the congestion-adaptive routing protocol (CRP) to prevent congestion. Simulation results show that CRP improves significantly the packet loss rate and the end-to-end delay compared to AODV and DSR. In [14], the authors introduced a type of service aware routing protocol (TSA) which uses both the packet type of service and the traditional hop count as route selection metrics. This proposal tries to avoid congestion by distributing the load over a potentially greater area. A linear load balancing protocol using multiple paths is proposed in [15], where *n* routes are sorted in increasing order of their hop count and have assigned priority values so that the shorter the path the higher the priority of the path to be selected to distribute the transmitted packets. In [16], the authors introduced a mechanism to find a primary forwarding route using the basic AODV engine and an alternative backup path to be used when the main route is broken. The backup path excludes nodes already used in the primary path, so that they are node-disjoint paths. The work [17] presented the Fibonacci multipath load balancing protocol (FMLB) for MANETs to distribute transmitted packets over multiple paths through the mobile nodes using an algorithm based on the Fibonacci sequence.
(c) Multipath routing protocols offer interesting benefits to increase the available bandwidth, which is suitable to transmit video over ad hoc networks where the bandwidth is a scarce resource. Several works such as [18–22] presented interesting approaches to transmit video flows over multipath frameworks. In [18], the authors analyzed the topic of multipath routing for multiple description video coding in wireless ad hoc networks. They found that genetic algorithms (GAs) are effective to address this type of cross-layer optimization problems. They demonstrate using numerical results the superior performance of their GA-based approach. In [19] the authors proposed an optimal routing algorithm to distribute video over multiple paths seeking to minimize congestion and to improve the video quality. In [20] the authors designed an adaptive multipath multimedia dynamic source routing (MMDSR) protocol able to self-configure dynamically depending on the state of the network while taking into account the special features of the video frames to distribute them over the multipath scheme. The authors in [21] proposed an extension of multipath optimized link state routing protocol (MP-OLSR) named fuzzy-based quality of service MP-OLSR (FQ-MP-OLSR) integrating two fuzzy systems. The first is used to calculate a multi-constrained QoS metric based on delay, throughput and signal to interference plus noise ratio (SINR) while the second is applied to adapt cost functions used to penalize paths previously computed by the Dijkstra's algorithm. Simulation results showed that FQ-MP-OLSR achieves a significant improvement in terms of QoS and quality of experience (QoE). The work in [22] presented a QoS-aware routing framework combining three QoS mechanisms, *i.e.*, cross-layer communication mechanism, session admission control and QoS-aware multipath routing to achieve an efficient video transmission over MANETs. Simulation results showed the improvement obtained in terms of video quality.
(d) Concerning the application of game theory in the routing for ad hoc networks, works in the literature deal mainly with two important issues: proposals that include incentives to *encourage nodes to cooperate* [23,24] and proposals that apply game theory *to attain a QoS-aware framework* [25–27]. In the case of MANET nodes that do not belong to a single authority, nodes do not have a common goal and seek to maximize their own utility trying to save their limited resources by reducing packet forwarding for others. Besides, mobility in MANETs causes hard challenges in the provision of the QoS required to distribute multimedia data. In [23], an analysis of cooperation incentives provided by reputation systems, price-based systems and an incentive strategy using game theory was presented. Also, the authors proposed an integrated system combining the previous strategies. Results clearly prove the benefits of the integrated system over the individual reputation system and the price-based system in terms of cooperation's effectiveness as well as in selfish node detection. In [24], the authors presented a novel incentive scheme for probabilistic routing which stimulates selfish nodes to participate. Results showed a 75.8% gain in delivery ratio compared to the case of a probabilistic routing providing no incentive. In [25], a dynamic probabilistic protocol based on game theory, called forwarding game routing protocol (FGRP), was introduced for the selection process of the forwarding nodes. In this protocol, a node is a player of the forwarding game and takes the forwarding decision upon the reception of a flooding packet. Each node tries to maximize its utility by selecting an appropriate strategy. Simulations show the benefits of FGRP in terms of end-to-end delay and packet delivery ratio. Current works in the literature that are similar to ours are [26,27]. In [26] the authors use game theory to design a self-optimizing algorithm to minimize the end-to-end delay in a multi-class MANET. The approach consists of a fully distributed algorithm based on AODV for which they analytically prove that it reaches an optimal routing for each user in terms of delay, and which also minimizes the overall delay in the network. They apply a potential game that meets the principle of *individual optimization provides a global optimal configuration*. They include a numerical evaluation that shows improvement compared to AODV. In our case, we use a multipath (instead of only one path) forwarding scheme to transmit real video (instead of Poisson traffic) and we use several metrics (instead of only using the delay) to arrange the paths. The work [27] studies the optimal forwarding problem in MANETs based on a generalized two-hop relay (the source node replicates copies of its packets to other relaying nodes so that each packet travels at most two hops to reach its destination) with limited packet redundancy *f* (*f*-cast, each packet can be replicated to at most *f* different relaying nodes) for packet routing. They propose a forwarding game where each node *i* individually decides a probability *p_i_* to send its own traffic and helps to forward other traffic with probability 1 − *p_i_*. The payoff for a node is the achievable throughput capacity of its own traffic. They obtain the optimal forwarding strategy that each node should adopt to ensure the optimum per node throughput capacity. Similarly to us they also mathematically derive a per node forwarding probability using game theory, although in their case it is used by a node to send/forward or not a packet, whereas in our case it is used by a node to decide if it sends a video frame through the best path or through the second best one. Besides, we use different forwarding schemes.

In this present work, we deal with the issue of QoS provision and leave cooperation encouragement for future work. After analyzing the multipath routing protocols presented in this section, for our purpose of distributing video messages over MANETs and VANETs, the most suitable approach as a reference are our previous proposals MMDSR [20] and g-MMDSR [3]. MMDSR is a QoS-aware self-configured multipath routing protocol that dynamically adapts to the changing environment, whereas g-MMDSR enhances MMDSR by including a 2-player game-theoretical algorithm in the forwarding scheme. To the best of our knowledge, the aim to apply together a QoS-aware multipath routing scheme and a multi-user game-theoretical approach that includes the particular features of the video frames into the game model to efficiently distribute video warning messages over ad hoc networks in smart cities is novel. In this work, the multipath routing scheme of MMDSR is enhanced by including a multi-user game-theoretical algorithm so that competing nodes share the scarce resources in a more suitable and efficient way. Our previous proposal [3] introduced a 2-player game-theoretical routing scheme to improve MMDSR, and in this present work we will develop a general multi-player game-theoretical routing protocol. In the next section, we summarize MMDSR in a nutshell.

## 3. Multipath Multimedia Dynamic Source Routing (MMDSR)

In this section, we give a brief summary of the main features of the MMDSR routing protocol, whose detailed description was presented in [3]. In this present article, we further improve the routing scheme by designing a novel game-theoretical model to provide video-streaming in MANETs. The framework is able to provide video-streaming services over IEEE 802.11e [28] MANETs and to dynamically adapt to the changing network conditions inherent in MANETs.

### 3.1. Basics of the General Framework

**Figure 1:**
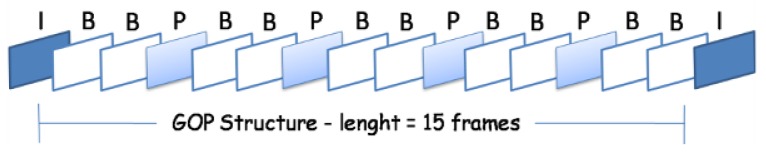
MPEG-2 GoP structure.

The multipath routing scheme of MMDSR is based on the DSR (Dynamic Source Routing) protocol [12] to find available paths from source to destination. Video is distributed using RTP/RTCP (Real-time Transport Protocol/Real-time Control Protocol) [29] over UDP as transport protocols. Our system uses a layered MPEG-2 VBR coding of the video flow, which is formed by sets of frames, usually 4 to 20 frames, called GoP (Groups of Pictures), see Figure 1. A GoP has three types of frames: I, P and B, and has a unique frame-pattern in a video repeated in each GoP. I (Intra) frames encode spatial redundancy, they form the base layer, provide a basic video quality and carry the most important information for the decoding process at the receiving side. The whole GoP would be lost if the corresponding I frame were not available at decoding time. P (Predicted) and B (Bi-directional) frames carry differential information from preceding (P) or preceding and posterior (B) frames, respectively. Considering these characteristics, we assign different priorities to the video frames according to their importance within the video flow. Therefore, I frames should have the highest priority, P frames the medium priority and B frames the lowest one.

In the MAC (Media Access Control) layer, we use the IEEE 802.11e [28] standard, which provides QoS support. It consists of four different Access Categories (AC), each with their own configuration parameters, *i.e.* contention window minimum (*CW_min_*) and maximum (*CW_max_*), arbitration interframe space (AIFS) and transmission opportunity (TxOp). Each packet from the higher layers arrives at the MAC layer with a specific priority value and is mapped into the proper AC. We defined the mapping of the different packets into each one of the four *AC_S_* as follows:

- AC0: signaling.
- AC1: high priority packets (I frames).
- AC2: medium priority packets (P frames).
- AC3: low priority packets (B frames + other best effort traffic).

### 3.2. Multipath Routing Scheme in MMDSR

**Figure 2:**
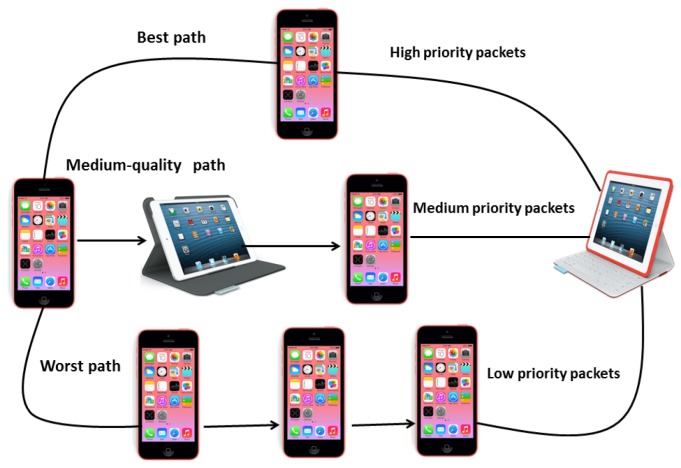
Multipath routing scheme using three paths.

MMDSR is a multipath routing protocol that uses the standard DSR as the engine to search for available paths. MMDSR uses up to three paths through which the three types of video frames will be sent. As Figure 2 shows, traditionally the most important video frames (I frames) would be sent through the best available path, P frames through the second best path and B frames through the third best path (worst one). In our results, we obtained that there are notable benefits in arranging two or three paths to transmit the video frames. However, arranging more than three paths in a multipath scheme does not provide a big improvement, while increases unnecessarily the management. Similar results were obtained in [30].

The user requirements are negotiated using QoS parameters to provide the required image quality. We use the following parameters: minimum expected bandwidth (*BW_min_*), maximum percentage of packet losses (*L_max_*), maximum delay (*D_max_*) and maximum delay jitter (*J_max_*).

(1) $$\text{user}\_\text{req}\equiv \{BW_{\min},L_{\max},D_{\max},J_{\max}\}$$

### 3.3. MMDSR Control Packets

**Figure 3:**
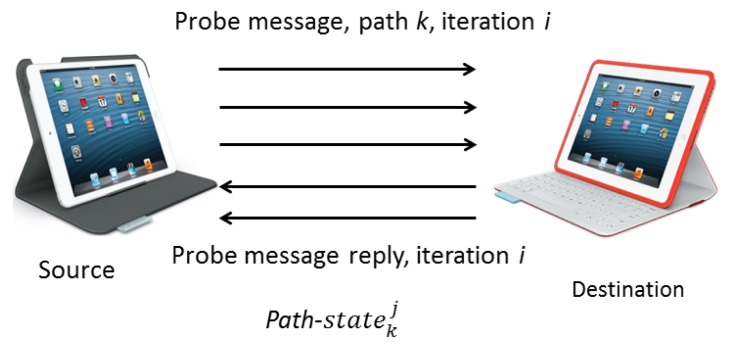
PM and PMR packets.

Decisions such as path selection or tuning of configuration parameters are operated from the source. MMDSR periodically discovers *AP* available paths between source and destination by sending monitoring *Probe Message* (PM) packets. After that, a *Probe Message Reply* (PMR) packet is generated at destination to carry the collected information about the quality of the available paths. The reduced size of these packets (around 64 bytes, depending on the number of paths found out) and the low frequency of sending them (every 3–18 s depending on the network state, see Equation (4)) makes the incurred overhead almost negligible. Figure 3 shows PM and PMR packets which are periodically interchanged between source and destination.

Then, a score is given to each one of the paths after analyzing the feedback information at the source node to classify them accordingly. That score is updated continuously after the reception of each PMR packet. Looking at the QoS parameters of the paths, the source selects three paths (if it is possible, or less if it is not) to compose the multipath scheme. The vector $\text{path}-{\text{state}}_{k}^{j}$ contains all the quality parameters calculated for each one of the available paths:

(2) $$\text{path}-{\text{state}}_{k}^{j}\equiv {\left\{BW,L,D,J,H,RM,MM\right\}}_{k}^{j}$$

where *j* is the iteration number of the algorithm and *k* refers to each one of the paths (with *k* ≤ *AP*). The QoS parameters are: available bandwidth ( $BW_{k}^{j}$), percentage of packet losses ( $L_{k}^{j}$), delay ( $D_{k}^{j}$), delay jitter ( $J_{k}^{j}$), hop distance ( $H_{k}^{j}$), reliability metric ( $RM_{k}^{j}$) calculated from the SNR (Signal to Noise Ratio) of the links involved in each path, and mobility metric $MM_{k}^{j}$ calculated from the relative mobility of the neighboring nodes within each path. The last two metrics were previously proposed in [3].

This process is repeated periodically to refresh the paths since the topology of MANETs vary and might produce link breakages. The routing period depends on the network state, as it is explained in Section 3.5.

### 3.4. Path Classification in MMDSR

Once the source has selected a set of paths that fulfil the requirements depicted in Equation (1), the classification of those paths is done by checking sequentially the qualifications of the QoS parameters, according to the following list:

$$RM_{k}^{j}+MM_{k}^{j},H_{k}^{j},BW_{k}^{j},L_{k}^{j}+J_{k}^{j}\;\text{and}\;D_{k}^{j}.$$

We first arrange the available paths looking at the metrics RM and MM, since we prefer the most reliable and stable paths to distribute video over MANETs. In case of draw, the decision is taken depending on the hop-count metric (*H*) which decides the shortest path. In case of another draw, we consider bandwidth (*BW*), then losses (*L*) and delay jitter (*J*), and finally delay (*D*). Nonetheless, other alternative algorithms to arrange the available paths could also be considered. Finally, the source selects the *k* paths (with *k* ≤ *AP*) required to compose the multipath routing scheme. In our case, *k* = 3 paths. Notice that if only two paths were available, we still could differentiate both paths (*i.e.*, the best and the medium-quality path), but if only one was available then all the packets would be sent through that single path.

### 3.5. MMDSR Self-Configuration

Due to the highly variable network topology of MANETs, any proposed routing protocol should be dynamic. Having this in mind, MMDSR is able to self-configure. Here, we will just point out the basics of the self-configuration operation.

Our framework monitors the current state of the network and in case of changes, the algorithm modifies the routing period of the algorithm and the thresholds to classify paths. We adjust those parameters dynamically depending on a parameter called *NState*, which brings information about the global network state and is updated by the algorithm iteration by iteration. *NState* is computed as follows:

(3) $$\begin{array}{cc} {\text{NState}}^{j} & =w_{RM}\cdot \bar{RM^{j}}+w_{MM}\cdot \bar{MM^{j}}+w_{BW}\cdot \bar{BW^{j}}+\\ & w_{L}\cdot \bar{L^{j}}+w_{D}\cdot \bar{D^{j}}+w_{J}\cdot \bar{J^{j}}+w_{H}\cdot \bar{H^{j}} \end{array}$$

In Equation (3), the upper bars denote averages and the *w*s are the weights assigned to each metric. When the source receives the feedback from the network by means of PMR packets, it calculates the *NState* using Equation (3) from the QoS parameters of all the *k* paths (see Equation (2)) on that itereation *j*.

The routing period (*T_routing_*) to refresh the multipath scheme also varies dynamically and is calculated according to Equation (4), so that when the network is behaving well (*i.e.*, *NState* is high), *T_routing_* is also high and the paths that form the multipath scheme will be used longer. Conversely, if the network is behaving bad (*i.e.*, *NState* is low) then *T_routing_* is low and the paths are refreshed sooner.

(4) $$T_{\text{routing}}^{j+1}=\gamma \cdot {\text{NState}}^{j}+\theta$$

To obtain the previous equation, a high number of simulations were conducted under a wide range of network conditions where the network performance was good, normal and bad. For the scenario under consideration (also used in the present paper), the obtained values were *γ* = 10 and *θ* = 3.

Till now, we have summarized the basics of the previous QoS-aware adaptive multipath routing protocol [3]. Next, we introduce our novel game-theoretical routing scheme to further improve the transmission of video over MANETs.

## 4. A Game-Theoretical Routing Protocol for MANETs

Game Theory is a branch of applied mathematics that has been used basically in economics to model competition between companies. During the last years, Game Theory has also been used in networking to solve routing and resource allocation problems in a competitive environment. MANET nodes take decentralized decisions, and resource management mechanisms can help those nodes to behave constructively improving the network performance as a whole [31]. In this work we apply Game Theory in the multipath routing protocol to further improve our proposal. We assume that each source node has a set of I, P and B video frames of a video flow to be transported; also, we assume that each source has three paths through which those frames could be sent. Nodes *play* a *routing game* to distribute the video flows seeking their own best performance. The *players* of the game are the MANET nodes and the *action* of the game is to select the proper route to forward their video-streams. In the following section, we will introduce the game-theoretical proposal included in the multipath routing scheme.

### 4.1. The Bases of Our Proposal

**Figure 4:**
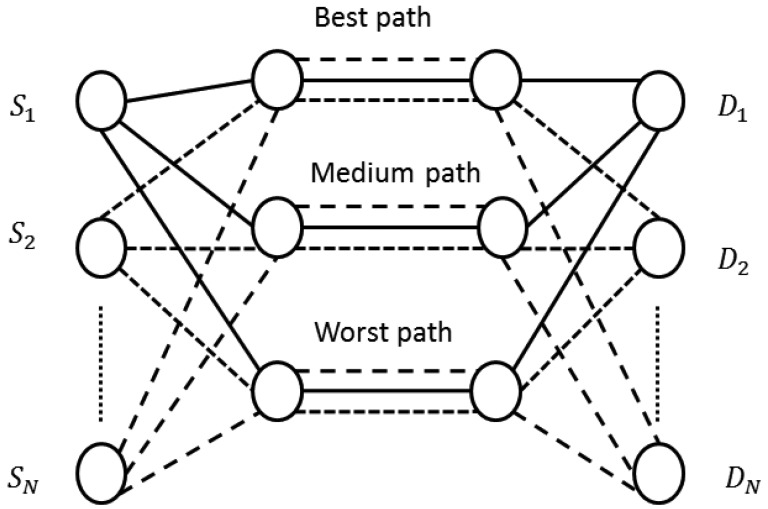
Proposed framework to send the video frames.

Figure 4 shows the proposed architecture. We assume *N* connections (*S*_1_-*D*_1_, *S*_2_-*D*_2_,…, *S_n_*-*D_n_*) and three paths. It is likewise possible to apply the proposal to any MANET independently of the number of connections, nodes and paths.

Traditionally, nodes always would try to send the most important video frames through the best available path obtained by the multipath routing protocol. This means that I frames, which are the biggest ones and carry the most important video information, will be sent through the best path, whereas the least important frames (*i.e.*, B frames) will be sent through the worst one. Nevertheless, if each node sends the most important frames through the best path, this path could get congested. As a consequence, that best path could suffer more losses than the others, which would lead to classify it as a worse path. This behavior could produce an oscillatory performance that might affect the video experience of users if it happened frequently.

To cope with this issue, users could *play a game* such that the best two paths (best, medium) could be selected by each player to transmit the most important video frames (*i.e.*, I+P frames). That is, each user could prefer to send sometimes the most important frames through the medium-quality path. Just for simplicity, B frames are considered always to be sent through the third path, which is the worst one. Also, I and P frames belonging to the same video stream are going to be sent through the same path to make more evident the inconveniences of sharing the same path, since there are more P frames than I frames per flow.

**Figure 5:**
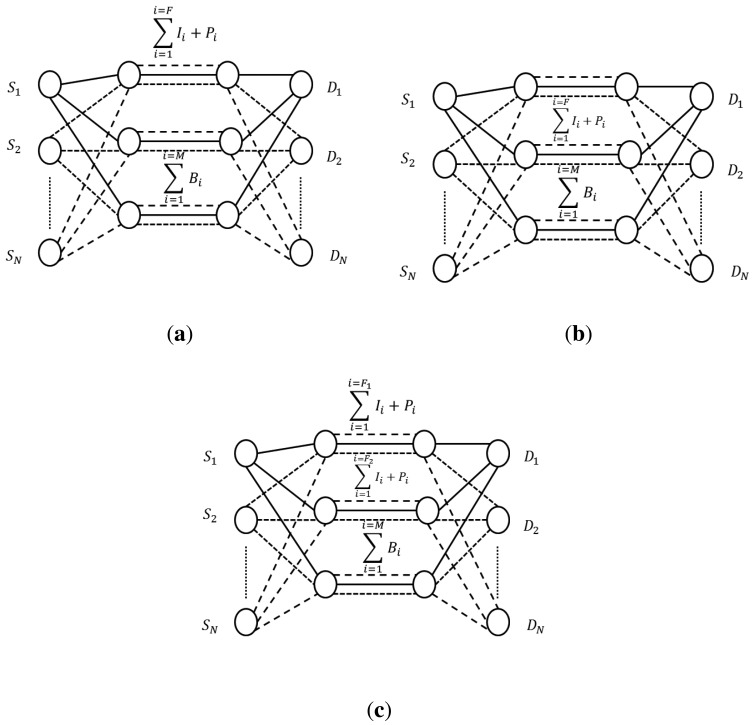
Three possible allocation situations after playing the game. *F* and *M* represent the number of (I+P) and B frames to be sent, respectively. All the B frames are always sent through the worst path. (**a**) All the I+P frames are sent through the best path; (**b**) All the I+P frames are sent through the medium-quality path; (**c**) I+P frames will be sent through the best path with a certain probability *p* and through the second best path with a probability *1-p*. *F*_1_ and *F*_2_ represent the number of (I+P) frames sent through the best path and the medium-quality path, respectively, being *F* = *F*_1_ *+ F*_2_.

In our game, in each iteration, users select paths for their respective video flows. As it is shown in Figure 5, we have three possible situations. Without playing the game, all users would always send the important frames through the best path (Figure 5a). Alternatively, they could play our routing game. Notice that case (Figure 5b) is worse than (Figure 5a) for all users since they are sending their frames together through the medium-quality path (this should not happen often). In the third case (Figure 5c), I+P frames will be sent through the best available path by each user with a certain probability *p* and through the second best path available with a probability 1 − *p*.

Notice that players (users) must decide their choices simultaneously and without communicating with each other. If we have a number of I+P frames equal to *F* to be sent, depending on the *p* value, a number of I+P frames equal to *F*_1_ will be sent through the best path and a number of I+P frames equal to *F*_2_ will be sent through the medium-quality one, being *F* = *F*_1_ + *F*_2_. *M* represents the number of B frames to be sent, always through the worst path.

In the next section we will compute the optimal probability *p* (called p*) of sending I+P frames through the best path that produces the best outcome for each player.

## 5. Game-Theoretical Routing Scheme for Video-Streaming in MANETs

A *game* can be described by listing the players participating in the game, a set of strategies for those players, and a specification of payoffs for each combination of strategies. Let *S* be a finite set of *N* players 1,…, *N*. Each player *i* has a finite set of available actions *A_i_*. Let *a_i_* ∈ *A_i_* be each particular action chosen by player *i*. The action space, *A*, is the cartesian product of all *A_i_*, *i.e.*, *A* = *A*_1_ × *A*_2_ × … *A_N_*. An *N*-tuple action, *a*, is a point in the action space *A*. A *pure strategy* provides a complete definition of how a player will play a game. In particular, it determines the move a player will make for any situation it could face. A *mixed strategy* of player *i*, *α_i_*, is an assignment of a probability, *p_i_* ∈ *P* = [0,1], to each pure strategy. This allows a player to randomly select among the set of pure strategies. Let *α* = (*α*_1_, *α*_2_,…, *α_N_*) be the mixed strategy profile, then the probability that a particular *N*-tuple action, *a* = (*a*_1_,*a*_2_,…,*a_N_*), will occur, *p*(*a*), is formed from the product of the probabilities assigned to *a* by *α*. Let *u_i_* be the *utility function* of player *i* in the strategic form game occurring in each stage. The utility function is a mathematical description of preferences that maps the action space to a set of real numbers. A utility function for a given player assigns a real number for every possible outcome of the game, so that a higher number implies that the outcome is more preferred.

(5) $$u_{i}:A\to R$$

*U_i_*(*α*) is the expected utility for player *i* for the mixed strategy profile *α*, and has the following expression:

(6) $$U_{i}(\alpha )\equiv \sum_{a\in A}p_{a}\cdot u_{i}(a)$$

A strategic game *G* can be expressed using three primary components: the set of players *S*, the action space *A*, and the set of individual utility functions for player *i*, *u_i_*.

(7) $$G=(S,A,u_{i})$$

A mixed strategies extension to *G* is given by the next expression, where Δ(*A_i_*) is the set of all probability distributions over *A_i_* and *U_i_* is the set of all expected utilities to *i*.

(8) $$G^{\prime }=(S,\Delta (A_{i}),U_{i})$$

A *best response* is a strategy which produces the most favorable outcome for a player, taking other players' strategies as given. A *Nash Equilibrium* (NE) [32] is a solution in which each player plays a best response to the strategies of other players. Each player is assumed to know the strategies of the other players, and no player has incentive to unilaterally change their current strategy while the other players keep theirs unchanged. Players are in equilibrium if a change in strategies by any one of them would lead that player to earn less than if they remained with their current strategy. It is a mathematical fact that every mixed extension of a strategic game has at least one *mixed strategy Nash equilibrium* [32].

To define best responses more generally, we need a notation for the set of strategies used by all players other than player *i*, named *α*_−_*_i_*:

(9) $$\alpha_{-i}=(\alpha_{1},\ldots ,\alpha_{i-1},\alpha_{i+1},\ldots ,\alpha_{N})$$

Strategy $\alpha_{i}^{*}$ is a best response for player *i* to the strategies of all players except *i*, $\alpha_{-i}^{*}$ if:

(10) $$U_{i}(\alpha_{i}^{*},\alpha_{-i}^{*})\geq U_{i}(\alpha_{i},\alpha_{-i}^{*}),\forall \alpha_{i}\in \Delta (A_{i})$$

This means that if $\alpha_{i}^{*}$ is a best response for player *i* to the assumed set of strategies $\alpha_{-i}^{*}$ played by the other *N-1* players, then it must give player *i* a payoff at least as large as the player would get if they placed any other strategy *α_i_* from their set of allowed strategies. Equivalently, a best response (BR) correspondence to player *i* is given by :

(11) $$\alpha_{i}^{*}\in BR_{i}(\alpha_{-i})={\text{argmax}}_{\alpha_{i}\in \Delta (A_{i})}U_{i}(\alpha_{i},\alpha_{-i})$$

Let us remark that *argmax_x_F*(*x*) is the value of *x* for which *F*(*x*) has the largest value. A joint strategy $\alpha^{*}=\left(\alpha_{1}^{*},\ldots ,\alpha_{N}^{*}\right)$ is a NE if, for each player *i*, $\alpha_{i}^{*}$ is a best response to $\alpha_{-i}^{*}$.

Remember that every finite game (finite number of players, each of which with a finite set of strategies) has Nash equilibria in either mixed or pure strategies [33,34].

### 5.1. The Benefit of Using a Particular Path to Transmit the I+P Video Frames

Before defining the player's utility of the game, we will define a parameter that evaluates the benefit of using a particular path. As we have mentioned before, we assume that we always have at least two available paths (best and medium-quality paths) to send packets. Each path will have its own benefit as we describe next.

**Definition:** *The benefit of a path_k_* is:

(12) $$\beta_{k}=\{\begin{array}{ll} \phi_{k}, & \text{if the path fulfills the Qos requirements}(\text{see Equation}(1))\\ 0, & \text{otherwise} \end{array}$$

where *ϕ_k_* ∈ ℝ*.

Let us assign *ϕ_k_* = *ϕ_b_* as the benefit for the best path and *ϕ_k_* = *ϕ_m_* as the benefit for the medium-quality path, where *ϕ_b_* and *ϕ_m_* ∈ ℝ*. Later, we will relate *ϕ_b_* and *ϕ_m_* with the subjective QoS experienced by the users regarding the video frames received from each path (see Equation (23)). Strategy *α_i_* is defined as follows:

(13) $$\alpha_{i}=\{\begin{array}{l} \text{Transmit using the best path}.\\ \text{Transmit using the medium-quality path}. \end{array}$$

Probability *p* is the probability of sending (I+P) frames through the best path and probability (1 − *p*) is the probability of sending those frames through the medium-quality path.

### 5.2. Design of the Utility Function

The utility function *U_i_* designed for our game-theoretical routing protocol aims at achieving two goals: Minimizing the rate of (I+P) frames lost and minimizing the delay jitter, since these two parameters are most significant in video-streaming services. An initial short delay can be easily bearable by the user. Part of that delay would be produced after gathering frames in the reception buffer to reorder frames before the decoding process. The proposed utility function for player *i* is the following:

(14) $$U_{i}=\underset{\text{Best path}}{\underset{︸}{\left(\frac{n_{rb,i}-n_{sb,i}}{n_{sb,i}}\right)\cdot \phi_{b,i}\cdot \frac{p_{i}^{2}}{J_{b,i}}}}+\underset{\text{Medium path}}{\underset{︸}{\left(\frac{n_{rm,i}-n_{sm,i}}{n_{sm,i}}\right)\cdot \phi_{m,i}\cdot \frac{{\left(1-p_{i}\right)}^{2}}{J_{m,i}}}}$$

All variables presented in Equation (14) are defined in Table 1.

Now let us relate *n_sb,i_* and *n_sm,i_* with *n_s,i_*:

(15) $$n_{sb,i}=p_{i}\cdot n_{s,i}$$

(16) $$n_{sm,i}=(1-p_{i})\cdot n_{s,i}$$

where *n_s,i_* = *n_sb,i_* + *n_sm,i_*.

Substituting Equations (15) and (16) in Equation (14) we get the following:

(17) $$U_{i}=\underset{\text{Best path}}{\underset{︸}{\left(\frac{n_{rb,i}-p_{i}\cdot n_{s,i}}{p_{i}\cdot n_{s,i}}\right)\cdot \phi_{b,i}\cdot \frac{p_{i}^{2}}{J_{b,i}}}}+\underset{\text{Medium path}}{\underset{︸}{\left(\frac{n_{rm,i}-(1-p_{i})\cdot n_{s,i}}{(1-p_{i})\cdot n_{s,i}}\right)\cdot \phi_{m,i}\cdot \frac{{\left(1-p_{i}\right)}^{2}}{J_{m,i}}}}$$

Notice that each player makes his/her own path classification, *i.e.*, each player might have different best and medium-quality paths.

In Equation (14), we have made our utility function *U_i_* to be proportional to the negative of the I+P frames losses. This way, the utility increases as the losses decrease, for both the best and the medium-quality paths. Besides, *U_i_* is a concave function so that we ensure to have a *p* value that produces the maximum utility. In Equation (14), $\left(\frac{n_{rb,i}-n_{sb,i}}{n_{sb,i}}\right)$ is the negative of the I+P frame losses through the best path and $\left(\frac{n_{rm,i}-n_{sm,i}}{n_{sm,i}}\right)$ is the negative of the I+P frame losses through the medium-quality path.

Besides, *U_i_* is proportional to the benefit of the path (*β_k_*, expressed in Equation (12)), that equals *ϕ_b_* and *ϕ_m_* for the best and the medium-quality path, respectively. Finally, *U_i_* is inversely proportional to the jitter delay, so that the utility increases as the jitter delay decreases. This is reflected as ( $\frac{1}{j_{b,i}}$) and ( $\frac{1}{J_{m,i}}$), for the best and the medium-quality paths, respectively. We can see a numerical example of the utility function in Section (7.2.3).

Depending upon the values of the utilities, pure strategies may not exist, but in that case there are always mixed strategies [33,34]. The mixed strategy $\alpha_{i}^{*}$ is a (NE) if the utilities *U_i_*(*i* = 1,…,*N*), satisfy Equation (11). If there exists a mixed Nash equilibrium, player *i* will have a best response. To obtain it, *U_i_* must be maximized:

(18) $$\frac{\partial U_{i}}{\partial p_{i}}=0$$

For the sake of a simpler writing we will omit the *i* index to refer the user in the previous variables shown in Table 1. Thus, we will use *n_sb_* instead of *n_sb,i_*, and so on.

Then, applying Equation (18) in Equation (17) we obtain:

(19) $$\frac{\partial U_{i}}{\partial p_{i}}=-2\cdot p_{i}\cdot \left(\frac{\phi_{b}}{J_{b}}+\frac{\phi_{m}}{J_{m}}\right)+\frac{\phi_{b}}{J_{b}}\cdot \frac{n_{rb}}{n_{s}}-\frac{\phi_{m}}{J_{m}}\cdot \frac{n_{rm}}{n_{s}}+2\cdot \frac{\phi_{m}}{J_{m}}$$

To simplify the previous equation, we assume that *J_b_*, *J_m_*, *n_sb_*, *n_sm_* and *n_s_* are greater than zero. This assumption has sense since a jitter equal to zero is very improbable and at least one frame should have been sent as well. We define the following variables:

(20) $${\hat{\phi }}_{b}=\frac{\phi_{b}}{J_{b}},{\hat{\phi }}_{m}=\frac{\phi_{m}}{J_{m}},{\hat{n}}_{b}=\frac{n_{rb}}{n_{s}},{\hat{n}}_{m}=\frac{n_{rm}}{n_{s}}$$

Next, we substitute Equation (20) in Equation (19) and we get:

(21) $$\frac{\partial U_{i}}{\partial p_{i}}=-2\cdot p_{i}\cdot ({\hat{\phi }}_{b}+{\hat{\phi }}_{m})+{\hat{\phi }}_{b}\cdot {\hat{n}}_{b}-{\hat{\phi }}_{m}\cdot {\hat{n}}_{m}+2\cdot {\hat{\phi }}_{m}$$

Then, by combining both Equations (18) and (21), we attain the solution for the best probability of sending (I+P) frames through the best path, that gives a NE in the utility function *U_i_*. This is called as the best response of the game. Thus, using $p_{i}^{*}$ to compute the probability of sending I+P frames through the best path, is a strategy which produces the most favorable outcome for player *i*, taking the other players' strategies as a given.

(22) $$p_{i}^{*}=\frac{{\hat{\phi }}_{b}\cdot {\hat{n}}_{b}+{\hat{\phi }}_{m}\cdot (2-{\hat{n}}_{m})}{2({\hat{\phi }}_{b}+{\hat{\phi }}_{m})}$$

This way, each player *i* will continuously update his/her best response $p_{i}^{*}$ using Equation (22). To do so, the user easily obtains from the RTCP feedback packets: the number of I+P frames received so far from the best and the medium-quality paths (*n_rb_* and *n_rm_*, respectively), the number of I+P frames sent so far (*n_s_*) and the jitter delay through the best and the medium-quality paths (*J_b_* and *J_m_*, respectively). In the next section, we explain how the user computes the benefits for the best and the medium-quality paths.

### 5.3. Paths' Benefits Computation

We have designed the value of the benefit of a path to be proportional to the subjective video quality perceived by the user regarding the video frames received from that path. In this work, we use the MOS (Mean Opinion Score) as a measure of the subjective QoS.

Accordingly, we define the following equations to compute the benefits of the best and medium-quality paths, *ϕ_b_* and *ϕ_m_*, respectively:

(23) $$\phi_{b}=k_{b}\cdot {\text{MOS}}_{b}\;\text{and}\;\phi_{m}=k_{m}\cdot {\text{MOS}}_{m}$$

where *k_b_* and *k_m_* are constants, [*k_b_*, *k_m_*] ∈ ℝ*.

Notice that *k_b_* and *k_m_* cannot equal zero because in this case, *ϕ_b_* and *ϕ_m_* would be zero too, which means that both paths would not satisfy the QoS parameters (see Equation (12)). The first condition for a path to be used by the sources is Equation (12); otherwise that path would not be taken into consideration.

Substituting Equation (23) in Equation (20), we get

(24) $${\hat{\phi }}_{b}=\frac{\phi_{b}}{J_{b}}=\frac{k_{b}\cdot {\text{MOS}}_{b}}{J_{b}}\;\text{and}\;{\hat{\phi }}_{m}=\frac{\phi_{m}}{J_{m}}=\frac{k_{m}\cdot {\text{MOS}}_{m}}{J_{m}}$$

Next, using Equation (24) in Equation (22) we have this expression for the best response probability, $p_{i}^{*}$ :

(25) $$p_{i}^{*}\frac{\left(\frac{k_{b}\cdot {\text{MOS}}_{b}}{J_{b}}\right)\cdot {\hat{n}}_{b}+\left(\frac{k_{m}\cdot {\text{MOS}}_{m}}{J_{m}}\right)\cdot (2-{\hat{n}}_{m})}{2\cdot \left(\frac{k_{b}\cdot {\text{MOS}}_{b}}{J_{b}}+\frac{k_{m}\cdot {\text{MOS}}_{m}}{J_{m}}\right)}$$

Let $\frac{k_{b}}{k_{m}}=k_{b/m}$. As *k_m_* is different from zero, we can divide the whole equation Equation (25) by *k_m_*. After substituting, we obtain the Nash Equilibrium strategy for player *i*:

(26) $$p_{i}^{*}(k_{b/m})=\frac{\left(\frac{k_{b/m}\cdot {\text{MOS}}_{b}}{J_{b}}\right)\cdot {\hat{n}}_{b}+\left(\frac{{\text{MOS}}_{m}}{J_{m}}\right)\cdot (2-{\hat{n}}_{m})}{2\cdot \left(\frac{k_{b/m}\cdot {\text{MOS}}_{b}}{J_{b}}+\frac{{\text{MOS}}_{m}}{J_{m}}\right)}$$

We designed *U_i_* to be a concave function, so that there is one p* value where *U_i_* is on its maximum value. Due to that, $\frac{\partial^{2}U_{i}}{\partial^{2}p_{i}}$ must be less than zero. This way, deriving Equation (21) we obtain:

(27) $$\frac{\partial^{2}U_{i}}{\partial^{2}p_{i}}=-2\cdot ({\hat{\phi }}_{b}+{\hat{\phi }}_{m})$$

As we need that $\frac{\partial^{2}U_{i}}{\partial^{2}p_{i}}<0$,

(28) $$\begin{array}{ccc} {\hat{\phi }}_{b}+{\hat{\phi }}_{m}>0; & \forall \;\left[\phi_{b},\phi_{m}\right]\in \mathbb{R}*, & [J_{b},J_{m}]\in {\mathbb{R}}_{+}^{*} \end{array}$$

Concluding, if player *i* adopts the strategy to send his/her (I+P) frames through the best path with a certain probability that equals $p_{i}^{*}$ (see Equation (26)), his/her own benefit and the whole benefit of the network will be the highest.

All values needed to compute $p_{i}^{*}$, except *k_b/m_*, can be obtained during normal network operation from the feedback information given by the RTCP packets. This way, users will update the probability $p_{i}^{*}$ with the current QoS parameters carried in the last received RTCP packet. Thus, *k_b_*_/_*_m_* is the single pending parameter to be obtained in Equation (26). In the next section, we will give a method to calculate analytically this parameter.

## 6. A Method to Calculate *k_b_*_/_*_m_*

**Figure 6:**
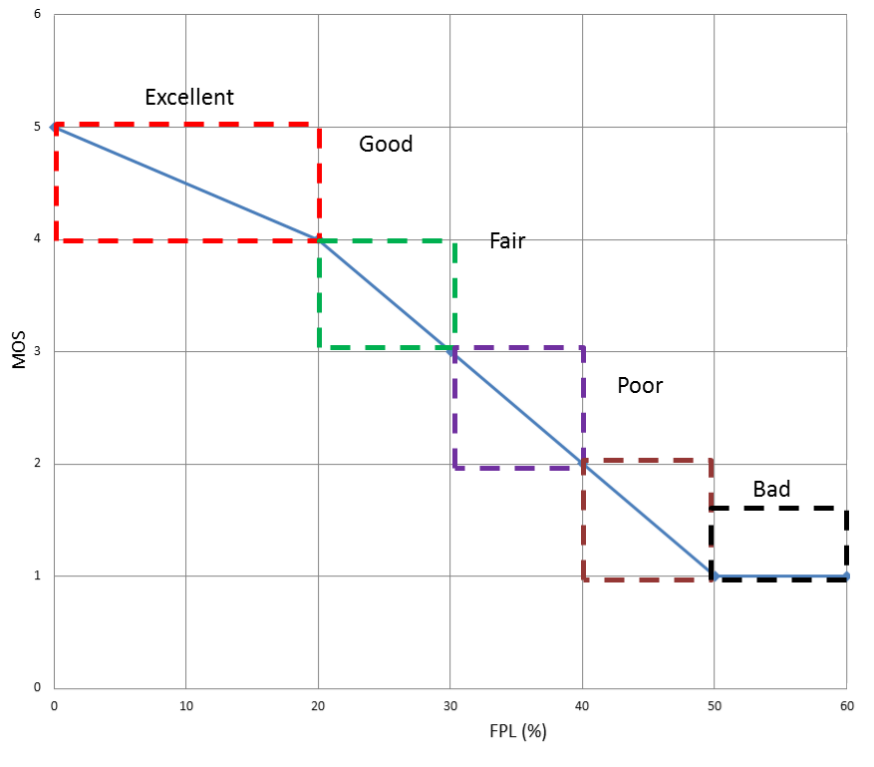
Subjective video quality measured by means of the mean opinion score (MOS) as a function of the fraction of packet losses (FPL).

*MOS_b_* and *MOS_m_* are the mean opinion score values measured in the best and the medium-quality path, respectively. They can take any value between 1 and 5, where 5 means *Excellent*, 4 means *Good*, 3 *Fair*, 2 *Poor* and 1 *Bad*. After making a video-streaming test, we obtained the following results shown in Table 2 and Figure 6. For further information about the test please see the Appendix A.

Now, our goal is to calculate *k_b/m_* so we can compute the value of $p_{i}^{*}$ using Equation (26). Three conditions will limit the computing of *k_b/m_*: 0 ≤ *p_i_* ≤ 1 and *U_i_* being a concave function. Below, we will study separately those three conditions.

### 6.1. Condition 1: *p_i_* ≥ 0

Combining Equation (22) with *p_i_* ≥ 0, we get that:

(29) $$\frac{{\hat{\phi }}_{b}\cdot {\hat{n}}_{b}+{\hat{\phi }}_{m}\cdot (2-{\hat{n}}_{m})}{2\left({\hat{\phi }}_{b}+{\hat{\phi }}_{m}\right)}\geq 0$$

Remember that Equation (28), which is the denominator of Equation (29) must also be fulfilled. Thus, we need:

(30) $${\hat{\phi }}_{b}\cdot {\hat{n}}_{b}+{\hat{\phi }}_{m}\cdot (2-{\hat{n}}_{m})\geq 0$$

After that, we substitute Equation (24) in Equation (30) to get the following first condition to be fulfilled by *k_b/m_*:

(31) $$\frac{k_{b}\cdot {\text{MOS}}_{b}\cdot {\hat{n}}_{b}}{J_{b}}+\frac{k_{m}\cdot {\text{MOS}}_{m}\cdot (2-{\hat{n}}_{m})}{J_{m}}\geq 0$$

Now, if we multiply the whole inequation Equation (31) by $\frac{1}{k_{m}}\left(\forall k_{m}\in {\mathbb{R}}^{+}\right)$ and rename $\frac{k_{b}}{k_{m}}$ by *k_b/m_*:

(32) $$\frac{k_{b/m}\cdot {\text{MOS}}_{b}\cdot {\hat{n}}_{b}}{J_{b}}+\frac{{\text{MOS}}_{m}\cdot (2-{\hat{n}}_{m})}{J_{m}}\geq 0$$

(33) $$\frac{k_{b/m}\cdot {\text{MOS}}_{b}\cdot {\hat{n}}_{b}}{J_{b}}\geq \frac{{\text{MOS}}_{m}\cdot ({\hat{n}}_{m}-2)}{J_{m}}$$

(34) $$k_{b/m}\geq \frac{{\text{MOS}}_{m}\cdot J_{b}\cdot ({\hat{n}}_{m}-2)}{{\text{MOS}}_{b}\cdot J_{m}\cdot {\hat{n}}_{b}}$$

### 6.2. Condition 2: *p_i_* ≤ 1

Combining Equation (22) with *p_i_* ≤ 1 leads to:

(35) $$\frac{{\hat{\phi }}_{b}\cdot {\hat{n}}_{b}+{\hat{\phi }}_{m}\cdot (2-{\hat{n}}_{m})}{2\cdot \left({\hat{\phi }}_{b}+{\hat{\phi }}_{m}\right)}\leq 1$$

Looking at Equation (28), we can write:

(36) $${\hat{\phi }}_{b}\cdot {\hat{n}}_{b}+{\hat{\phi }}_{m}\cdot (2-{\hat{n}}_{m})\leq 2\cdot \left({\hat{\phi }}_{b}+{\hat{\phi }}_{m}\right)$$

Now, we substitute Equation (24) in Equation (36) to get:

(37) $$\frac{k_{b}\cdot {\text{MOS}}_{b}\cdot {\hat{n}}_{b}}{J_{b}}+\frac{k_{m}\cdot {\text{MOS}}_{m}\cdot (2-{\hat{n}}_{m})}{J_{m}}\leq 2\cdot \left(\frac{k_{b}{\text{MOS}}_{b}}{J_{b}}+\frac{k_{m}\cdot {\text{MOS}}_{m}}{J_{m}}\right)$$

Again, we multiply the whole inequation by $\frac{1}{k_{m}}\left(\forall k_{m}\in {\mathbb{R}}^{+}\right)$ and rename $\frac{k_{b}}{k_{m}}$ by *k_b/m_*:

(38) $$\frac{k_{b/m}\cdot {\text{MOS}}_{b}\cdot {\hat{n}}_{b}}{J_{b}}+\frac{{\text{MOS}}_{m}\cdot (2-{\hat{n}}_{m})}{J_{m}}\leq 2\cdot \left(\frac{k_{b/m}\cdot {\text{MOS}}_{b}}{J_{b}}+\frac{{\text{MOS}}_{m}}{J_{m}}\right)$$

and removing $2\cdot \left(\frac{{\text{MOS}}_{m}}{J_{m}}\right)$ from both sides, we reach to:

(39) $$\frac{k_{b/m}\cdot {\text{MOS}}_{b}\cdot ({\hat{n}}_{b}-2)}{J_{b}}\leq \frac{{\text{MOS}}_{m}\cdot {\hat{n}}_{m}}{J_{m}}$$

Now, before we continue we will find out which is the sign of the expression (*n̂_b_* − 2). *n̂_b_* was defined in Equation (20) as the relation between the number of I+P frames received from the best path (*n_rb_*) and the total number of I+P frames sent (*n_s_*).

(40) $${\hat{n}}_{b}=\frac{n_{rb}}{n_{s}}=\frac{n_{rb}}{n_{sb}+n_{sm}}\leq 1$$

since *n_rb_* ≤ *n_sb_* (some I+P frames might have been lost through the best path) and *min*(*n_sm_*) = 0 (that minimum value is obtained when no frame was sent through the medium-quality path).

Therefore,

(41) $${\hat{n}}_{b}-2\leq -1$$

Finally, the second inequation to be fulfilled by *k_b/m_* is:

(42) $$k_{b/m}\geq \frac{{\text{MOS}}_{m}\cdot J_{b}\cdot {\hat{n}}_{m}}{{\text{MOS}}_{b}\cdot J_{m}\cdot ({\hat{n}}_{b}-2)}$$

### 6.3. Condition 3: Concave Function *U_i_*

For *U_i_* to be a concave function, Equation (28) must be fulfilled. Substituting Equation (24) in Equation (28), we get:

(43) $$\frac{k_{b}\cdot {\text{MOS}}_{b}}{J_{b}}+\frac{k_{m}\cdot {\text{MOS}}_{m}}{J_{m}}>0$$

If we multiply the whole inequation by $\frac{1}{k_{m}}\left(\forall k_{m}\in {\mathbb{R}}^{+}\right)$ and rename $\frac{k_{b}}{k_{m}}$ by *k_b/m_*, we get:

(44) $$\frac{k_{b/m}\cdot {\text{MOS}}_{b}}{J_{b}}>-\frac{{\text{MOS}}_{m}}{J_{m}}$$

Finally, we obtain Equation (45) as the third condition to be fulfilled by *k_b/m_*:

(45) $$k_{b/m}>\frac{-{\text{MOS}}_{m}\cdot J_{b}}{{\text{MOS}}_{b}\cdot J_{m}}$$

### 6.4. The Three Inequations to Be Fulfilled by *k_b_*_/_*_m_*

We first rewrite the three inequations to be satisfied by *k_b/m_*: Equations (34), (42) and (45). Besides, we will rename the thresholds of the three inequations as *α*_0_, *α*_1_ and *α*_2_, respectively

(46) $$\begin{array}{rr} k_{b/m}>\frac{-{\text{MOS}}_{m}\cdot J_{b}}{{\text{MOS}}_{b}\cdot J_{m}} & =\alpha_{0}\\ k_{b/m}\geq \frac{{\text{MOS}}_{m}\cdot J_{b}\cdot ({\hat{n}}_{m}-2)}{{\text{MOS}}_{b}\cdot J_{m}\cdot {\hat{n}}_{b}} & =\alpha_{1}\\ k_{b/m}\geq \frac{{\text{MOS}}_{m}\cdot J_{b}\cdot {\hat{n}}_{m}}{{\text{MOS}}_{b}\cdot J_{m}\cdot ({\hat{n}}_{b}-2)} & =\alpha_{2} \end{array}$$

**Figure 7:**
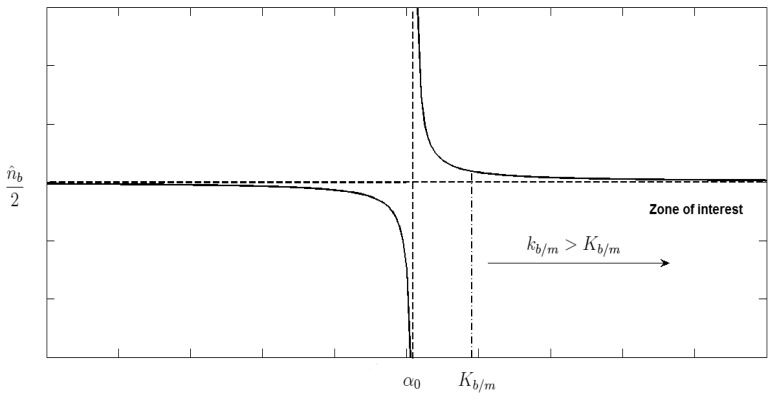
Best response probability $p_{i}^{*}$ as a function of *k_b/m_*, see Equation (26).

We need to find a value for *k_b/m_* that satisfies the three inequations. First of all, the range of solutions for *k_b/m_* is ]*K_b/m_*, +∞), where *K_b/m_* will be the maximum value among *α*_0_, *α*_1_ and *α*_2_. The probability $p_{i}^{*}\left(k_{b/m}\right)$ of sending (I+P) frames through the best path is depicted in Figure 7. The limit of $p_{i}^{*}\left(k_{b/m}\right)$ when *k_b/m_* → *∞* (horizontal asymptote) can be obtained from Equation (26) and it has the following value:

(47) $$\underset{k_{b/m}\to \infty }{\lim}p_{i}^{*}(k_{b/m})=\frac{{\hat{n}}_{b}}{2}$$

The vertical asymptote occurs at *k_b/m_*-value that makes the denominator zero (*i.e.*, *k_b/m_* = *α*_0_). We should find a value for *k_b/m_* in the range ]*K_b/m_*, +∞) with which $p_{i}^{*}\left(k_{b/m}\right)$ changes softly throughout time. This way, the transition in the selection between the best and the medium-quality paths will be smooth producing a more stable system. For that, we calculate the first derivative $\frac{\partial p_{i}^{*}\left(k_{b/m}\right)}{\partial k_{b/m}}$, which represents the slope value for each *k_b/m_* > *K_b/m_* (*i.e.*, Zone of interest).

(48) $$\frac{\partial p_{i}^{*}(k_{b/m})}{\partial k_{b/m}}=\frac{\left(\frac{{\hat{n}}_{b}\cdot {\text{MOS}}_{b}}{J_{b}}\right)\cdot \left(\frac{2\cdot k_{b/m}\cdot {\text{MOS}}_{b}}{J_{b}}+\frac{2\cdot {\text{MOS}}_{m}}{J_{m}}\right)}{4\cdot {\left(\frac{k_{b/m}\cdot {\text{MOS}}_{b}}{J_{b}}+\frac{{\text{MOS}}_{m}}{J_{m}}\right)}^{2}}-\frac{\left(\frac{2\cdot {\text{MOS}}_{b}}{J_{b}}\right)\cdot \left(\frac{k_{b/m}\cdot {\text{MOS}}_{b}\cdot {\hat{n}}_{b}}{J_{b}}+\frac{{\text{MOS}}_{m}\cdot (2-{\hat{n}}_{m})}{J_{m}}\right)}{4\cdot {\left(\frac{k_{b/m}\cdot {\text{MOS}}_{b}}{J_{b}}+\frac{{\text{MOS}}_{m}}{J_{m}}\right)}^{2}}$$

After simplifying the equation, we obtain:

(49) $$\frac{\partial p_{i}^{*}}{\partial k_{b/m}}=\frac{\frac{{\text{MOS}}_{b}}{J_{b}}\cdot \frac{{\text{MOS}}_{m}}{J_{m}}\cdot ({\hat{n}}_{b}+{\hat{n}}_{m}-2)}{2\cdot {\left(\frac{k_{b/m}\cdot {\text{MOS}}_{b}}{J_{b}}+\frac{{\text{MOS}}_{m}}{J_{m}}\right)}^{2}}$$

Now, we isolate *k_b/m_* from Equation (49) in terms of $\frac{\partial p_{i}^{*}\left(k_{b/m}\right)}{\partial k_{b/m}}$ and we get the following expression:

(50) $$k_{b/m}=\frac{\pm \sqrt{\frac{\frac{{\text{MOS}}_{b}}{J_{b}}\cdot \frac{{\text{MOS}}_{m}}{J_{m}}\cdot ({\hat{n}}_{b}+{\hat{n}}_{m}-2)}{2\cdot \frac{\partial p_{i}^{*}(k_{b/m})}{\partial k_{b/m}}}}-\frac{{\text{MOS}}_{m}}{J_{m}}}{\frac{{\text{MOS}}_{b}}{J_{b}}}$$

Here, we can see that (*n̂_b_* + *ĥ_m_*–2) < 0, since ${\hat{n}}_{b}=\frac{n_{rb}}{n_{s}}=\frac{n_{rb}}{n_{sb}+n_{sm}}\leq 1$, and ${\hat{n}}_{m}=\frac{n_{rm}}{n_{s}}=\frac{n_{rm}}{n_{sb}+n_{sm}}\leq 1$. Look at Equation (40) to see the easy justification for both expressions.

Consequently, we need that $\frac{\partial p_{i}^{*}}{\partial k_{b/m}}\leq 0$ to compute a proper *k_b/m_* value.

The parameters of Equation (50) that can be calculated during operation time are: the number of I+P frames received from the best path (*n_rb_*) and the number of I+P frames sent through the best path (*n_sb_*) to compute ${\hat{n}}_{b}=\frac{n_{rb}}{n_{sb}}$; the number of I+P frames received from the medium-quality path (*n_rm_*) and the number of I+P frames sent through the medium-quality path (*n_sm_*) to compute ${\hat{n}}_{m}=\frac{n_{rm}}{n_{sm}}$; the jitter delay through the best and the medium-quality paths (*J_b_* and *J_m_*, respectively) computed from the RTCP packets; and the MOS of the best and the medium-quality paths, computed with Equation (B1) presented in the Appendix B.

The only variable in Equation (50) that is not defined yet is $\frac{\partial p_{i}^{*}\left(k_{b/m}\right)}{\partial k_{b/m}}$. To design a proper value for $\frac{\partial p_{i}^{*}\left(k_{b/m}\right)}{\partial k_{b/m}}$, we carried out a high amount of simulations under different network conditions and with different values of $\frac{\partial p_{i}^{*}\left(k_{b/m}\right)}{\partial k_{b/m}}$ and we noticed that with a value of $\frac{\partial p_{i}^{*}\left(k_{b/m}\right)}{\partial k_{b/m}}=-0.2$, the variation of $p_{i}^{*}$ throughout time was soft without sharp changes. From Equation (50), we see that we have two possible values for *k_b/m_*, one of them is higher than *K_b/m_* and the other one is lower than *K_b/m_*, so we take the one which belongs to the range ]*K_b/m_*, +∞).

To conclude with, Algorithm 1 summarizes the methodology to compute the best response probability $p_{i}^{*}$ for player *i* to send his/her I+P frames through the best path.



**Algorithm 1** Calculation of 
$p_{i}^{*}$, the best response probability for player *i* that maximizes his/her utility function *U_i_*.
**Require:** Obtain updated QoS values from the periodically received RTCP packets.1:Obtain the values of (*MOS_b_*, *MOS_m_*, *J_b_*, *J_m_*, *n̂_b_*, *n̂_m_*)2:Compute the *k_b/m_* parameter designed that fulfills the requirements.
$k_{b/m}=\frac{\sqrt[{\pm }]{\frac{\frac{{\text{MOS}}_{b}}{J_{b}}\cdot \frac{{\text{MOS}}_{m}}{J_{m}}\left({\hat{n}}_{b}+{\hat{n}}_{m^{-2}}\right)}{2\cdot \frac{\partial p_{i}^{*}}{\partial k_{b/m}}}-\frac{{\text{MOS}}_{m}}{J_{m}}}}{\frac{{\text{MOS}}_{b}}{J_{b}}}$3:Calculate the probability 
$p_{i}^{*}$ to send I+P frames through the best path, that maximizes the utility function *U_i_*
$p_{i}^{*}\left(k_{b/m}\right)=\frac{\left(\frac{k_{b/m}\cdot {\text{MOS}}_{b}}{J_{b}}\right)\cdot {\hat{n}}_{b}+\left(\frac{{\text{MOS}}_{m}}{J_{m}}\right)\cdot \left(2-{\hat{n}}_{m}\right)}{2\cdot \left(\frac{k_{b/m}\cdot {\text{MOS}}_{b}}{J_{b}}+\frac{{\text{MOS}}_{m}}{J_{m}}\right)}$


## 7. Simulation Results

In this section, we first depict a case study in a smart city about which we will set the NS2 simulation scenarios. The case study involves an emergency situation in MANETs and VANETs to transmit a multimedia warning message to the closest emergency unit and to alert other citizens around. After that, we present a performance evaluation in a MANET scenario in Subsection 7.2, and in a VANET scenario in Subsection 7.3.

### 7.1. A Case Study in a Smart City

We focus this research work on two realistic smart city scenarios. A mobile adhoc network (MANET) and a vehicular ad hoc network (VANET), where emergency prevention and response are key issues. In the two scenarios under consideration, we assume that in a given moment an accident happened. Most of the citizens nowadays carry mobile phones or tablets. In the MANET/VANET scenario, we assume that a smart citizen/driver witnesses the situation, makes a short video-warning message (the driver will just push a button that will make a small exterior car-mounted camera shoot the video) about the accident and sends it through the MANETAANET to the nearest emergency unit (e.g., police, ambulances, hospitals). Authorities will respond upon receiving the video and will take proper actions. This way, with a video-warning message the emergency can be evaluated much better than with a simple text. It would be easier to ensure an accurate interpretation of the situation and the accident could be treated with the adequate level of seriousness. The smart citizen/driver sends a multimedia message which includes different information regarding the incident, e.g., the GPS location, a voice message and a short video of the incident. A suitable kind of smart-911 (112 in Europe) application in the citizen's mobile/vehicle sends the multimedia message to the smart-911 emergency center, who manages the proper actions for that incident. For instance, ambulances and paramedical will be sent there, traffic lights will turn to red around the accident, a green wave of traffic lights will help the ambulances get there sooner, the nearest hospital is warned, the doctors wait for the injuries, *etc*. A video of the incident facilitates a preliminary evaluation of the wounded people as well as helps to better determine the requirements needed to manage the dangerous situation. Our purpose in this work is to design a game-theoretical multipath routing protocol suitable to transmit those video-warning messages over MANETs/VANETs in this kind of smart city scenarios. In the next section, a detailed performance evaluation in the MANET scenario will be studied, whereas a first brief performance evaluation in a VANET scenario is done in Section 7.3.

### 7.2. Performance Evaluation in a MANET Scenario

We implemented our proposal in the open source network simulator NS2 [35] where we conducted simulations to evaluate the benefits of our approach. The MANET scenario was generated with the Bonnmotion tool [36]. The same quantity of interfering CBR traffic was generated in each simulation to constrain the paths. For instance, for 2 players, we generated interfering CBR traffic at 300 Kbps, 200 Kbps for 3 players, 150 kbps for 4 players and 120 kbps for 5 players. This way, a total interference CBR raffic of 600 Kbps was sent whatever the number of players was. The simulation settings of the scenario are shown in Table 3. All the figures show confidence intervals (CI) of 90 percent obtained from 20 simulations per point, each simulation with an independent Bonnmotion scenario.

The scenario used to test the proposal consists of a set of 50 mobile nodes distributed in a MANET of 520 × 520 m^2^. The transmission range of the nodes is 120 m. Nodes move with a speed up to 2 m/s. Video flows are transmitted from nodes *S_i_* to nodes *D_i_*, 1 ≤ *i* ≤ *N* where *N* is the number of players (sources). The paths discovered by the MMDSR routing protocol are classified by each user using the MMDSR path classification described in Section 3.4. Each source decides the path to route packets according to the game-theoretical routing algorithm presented in Section 5 and depicted in Figure 5.

**Figure 8:**
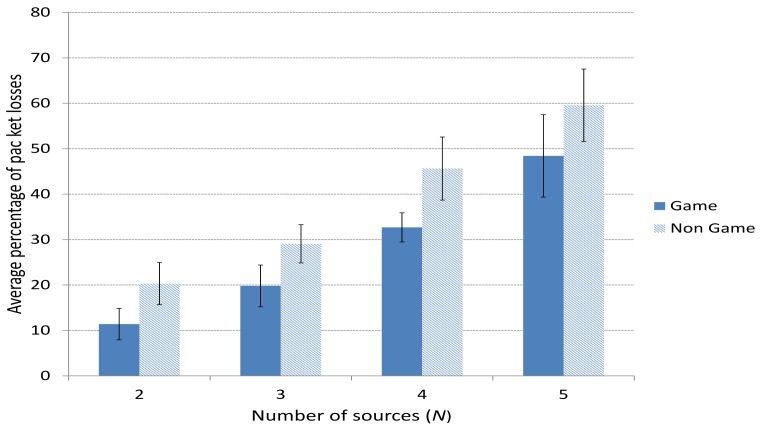
Average percentage of packet losses.

**Figure 9:**
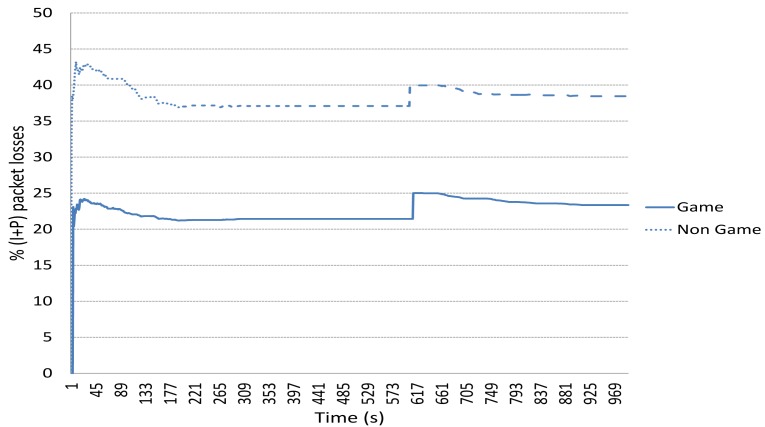
Percentage of packet losses vs time (*N* = 3 users).

Figure 8 shows the average percentage of packet losses when using the game-theoretical scheme for a variable *p* value calculated using Algorithm 1 against the case of non using it (Non game option). We can clearly notice how including the game-theoretical routing scheme, the average video packet losses are reduced around 10% for *N* = 2 to 5 users. The average packet losses decreases due to the optimal selection of paths based on a probability value (*i.e.*, p*) that optimally balances the load among the two paths at stake (*i.e.*, the best and the medium-quality paths). Figure 9 depicts the (I+P) packet losses throughout simulation time. In this case, we see how using the game-theoretical model, losses are around 20% lower than non using it.

**Figure 10:**
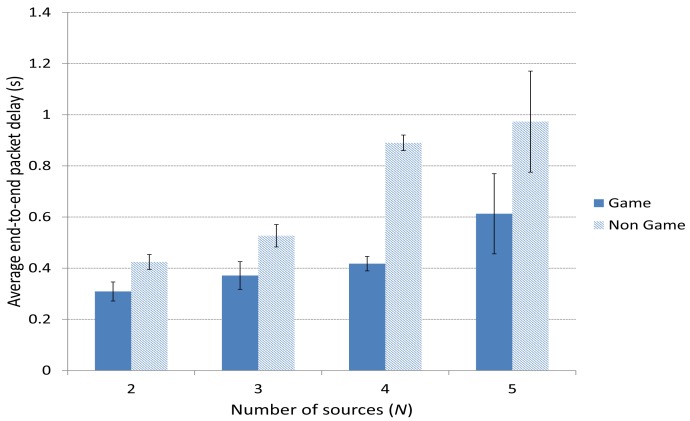
Average end-to-end packet delay.

Figure 10 depicts the average end-to-end packet delay. We can see that the case including the Game scheme improves the delay compared to the Non Game case. This is specially notable (around half a second) for a high number of sources *N* = 4 or 5, since in those cases, the amount of traffic is much higher and a smart selection of the forwarding paths gains importance.

**Figure 11:**
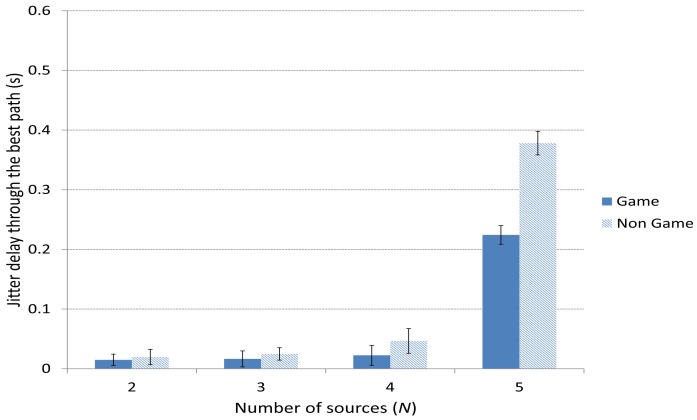
Delay jitter through the best path.

**Figure 12:**
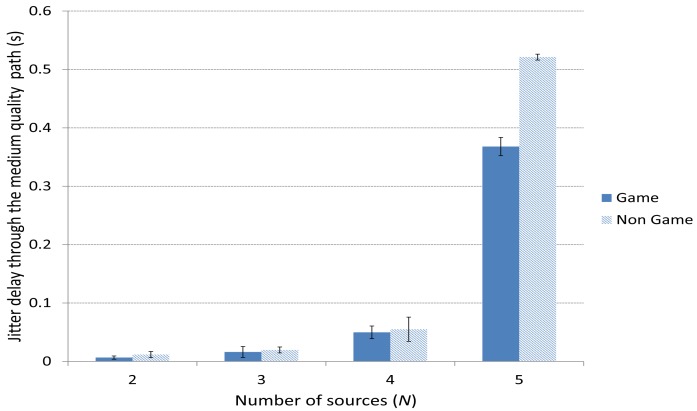
Delay jitter through the medium-quality path.

Figures 11 and 12 depict the average delay jitter suffered by packets through the best and the medium-quality path, respectively. In both cases, the jitter using the game-theoretical scheme shows a slightly better result against the Non Game case for 2 to 4 players. The improvement is notably higher for 5 sources, when the jitter is also higher due to the high traffic that produces a higher variation in the packet delays.

**Figure 13:**
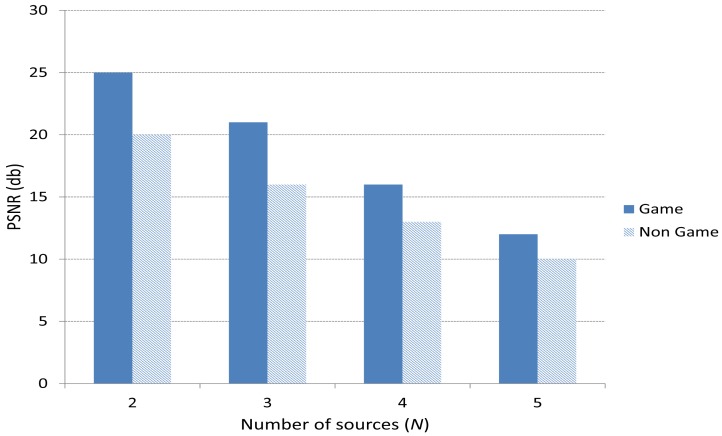
Peak Signal to Noise Ratio (PSNR).

Figure 13 depicts the peak signal-to-noise ratio (PSNR) obtained for 2, 3, 4 and 5 players. We can see that the case including the Game scheme improves the PSNR compared to the Non Game case. Also, PSNR decreases as *N* increases. This is because as *N* increases, the number of video frames to be sent increases, causing more packet losses and as a result a lower PSNR.

#### 7.2.1. Gain for I and P Frames

To better see separately the gain obtained for I and P frames, we define the following parameters:

(51) $$\%{\text{GainI}}_{i}=\left(\frac{{\text{ILNG}}_{i}-{\text{ILG}}_{i}}{{\text{ILNG}}_{i}}\right)\cdot 100$$

(52) $$\%{\text{GainP}}_{i}=\left(\frac{{\text{PLNG}}_{i}-{\text{PLG}}_{i}}{{\text{PLNG}}_{i}}\right)\cdot 100$$

where

- *i*: 2, 3, 4 and 5 sources (players).
- *ILNG_i_*: percentage of packet losses for I frames when the game-theoretical scheme is not used.
- *ILG_i_*: percentage of packet losses for I frames when the game-theoretical scheme is used.
- *PLNG_i_*: percentage of packet losses for P frames when the game-theoretical scheme is not used.
- *PLG_i_*: percentage of packet losses for P frames when the game-theoretical scheme is used.
- *GainI_i_*: Gain obtained for I frames using the game-theoretical scheme with respect to not using it.
- *GainP_i_*: Gain obtained for P frames using the game-theoretical scheme with respect to not using it.

**Figure 14:**
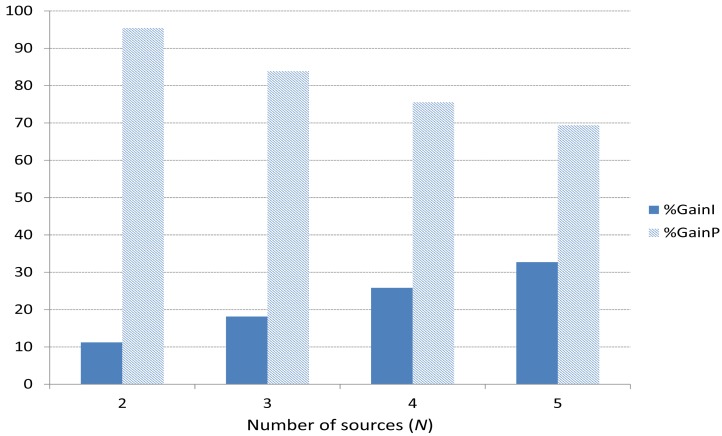
Gain in terms of packet losses for I and P video frames using the game-theoretical scheme with respect to not using it.

Figure 14 shows the average of all the simulation results for %*GainP_i_* and %*GainI_i_*. We can see that when the game-theoretical scheme is applied, the gain is 11%, 18%, 25%, 32% for I packets (dark bars) and 95%, 83%, 75%, 69% for P packets (light bars), for 2, 3, 4 and 5 players, respectively. As we can observe, %*GainP_i_* > %*GainI_i_* in all the cases. This is because there are much more P frames than I frames (see Figure 1) per player so the benefits are more noticeable for P frames. When *N* increases, the difference is lower, since the number of I frames increases.

In addition, we observe a slightly increment for %*GainI_i_* (dark bars) as *N* increases. This is because as *N* increases, the number of I frames increases and this makes the game-theoretical scheme benefits more noticeable. On the other hand, %*GainP_i_* decreases as *N* increases. The reason is that as *N* increases, there are too many P frames (more collisions), higher loads to be balanced and as a result we obtain a lower benefit. Nonetheless, %*GainP_i_* is still high (around 70% for 5 sources).

#### 7.2.2. Utility Function Values

In this section, we will compute the gain of our game-theoretical routing scheme. Let us define *U_G_i__* as the utility function for player *i* when the game-theoretical scheme is used and *U_NG_i__* as the utility function when it is not used. Both utility function values will be computed using Equation (17). *G_i_* is the gain obtained for player *i* by using the game-theoretical scheme with respect to not using it, 0 ≤ *G_i_* ≤ 1.

(53) $$G_{i}=\frac{U_{G_{i}}-U_{NG_{i}}}{U_{G_{i}}}=\frac{U_{p=p_{i}^{*}}-U_{p=1}}{U_{p=p_{i}^{*}}}$$

Using Equation (17) in Equation (53) we obtain:

(54) $$G_{i}=\frac{U_{G_{i}}-U_{NG_{i}}}{U_{G_{i}}}=\frac{\left(\frac{n_{rb,i}-n_{s,i}\cdot p_{i}^{*}}{n_{s,i}\cdot p_{i}^{*}}\right)\cdot \phi_{b,i}\cdot \frac{{\left(p_{i}^{*}\right)}^{2}}{J_{b,i}}+\left(\frac{n_{rm,i}-n_{s,i}\cdot (1-p_{i}^{*})}{n_{s,i}\cdot (1-p_{i}^{*})}\right)\cdot \phi_{m,i}\cdot \frac{{\left(1-p_{i}^{*}\right)}^{2}}{J_{m,i}}-\left(\frac{n_{rb,i}-n_{s,i}\cdot 1}{n_{s,i}\cdot 1}\right)\cdot \phi_{b,i}\cdot \frac{1}{J_{b,i}}}{\left(\frac{n_{rb,i}-n_{s,i}\cdot p_{i}^{*}}{n_{s,i}\cdot p_{i}^{*}}\right)\cdot \phi_{b,i}\cdot \frac{{\left(p_{i}^{*}\right)}^{2}}{J_{b,i}}+\left(\frac{n_{rm,i}-n_{s,i}\cdot (1-p_{i}^{*})}{n_{s,i}\cdot (1-p_{i}^{*})}\right)\cdot \phi_{m,i}\cdot \frac{{\left(1-p_{i}^{*}\right)}^{2}}{J_{m,i}}}$$

#### 7.2.3. A Numerical Example

In this section, we show a numerical example to calculate the gain obtained with our proposal for *N* = 2 users using Equation (54), *i.e.*, *G*_1_ and *G*_2_. To do that, we use the values obtained during simulation from the RTCP packets. They are shown in Table 4.

We calculate the variable *k_b/m_*_,1_ and *k_b/m_*_,2_ for players 1 and 2 using Equation (50) and the simulation output values shown in Table 4. Results are shown in Table 5.

After that, we simplify $\left(\frac{n_{rb,i}-n_{sb,i}}{n_{sb,i}}\right)$ and $\left(\frac{n_{rm,i}-n_{sm,i}}{n_{sm,i}}\right)$ as seen in Equations (55) and (56) in order to be easily calculated later using Table 4.

(55) $$\left(\frac{n_{rb,i}-n_{sb,i}}{n_{sb,i}}\right)=\frac{n_{rb,i}-n_{s,i}\cdot p_{i}^{*}}{n_{s,i}\cdot p_{i}^{*}}=\frac{{\hat{n}}_{b,i}}{p_{i}^{*}}-1$$

(56) $$\left(\frac{n_{rm,i}-n_{sm,i}}{n_{sm,i}}\right)=\frac{n_{rm,i}-n_{s,i}\cdot (1-p_{i}^{*})}{n_{s,i}\cdot (1-p_{i}^{*})}=\frac{{\hat{n}}_{m,i}}{\left(1-p_{i}^{*}\right)}-1$$

Next, we calculate *ϕ_b_* and *ϕ_m_* for each player using Equation (23) so they can be substituted in Equation (17).

(57) $$\phi_{b,1}=k_{b,1}\cdot MOS_{b,1}=k_{b/m,1}\cdot k_{m,1}\cdot MOS_{b,1}=3.134\cdot k_{m,1}$$

(58) $$\phi_{b,2}=k_{b,2}\cdot MOS_{b,2}=k_{b/m,2}\cdot k_{m,2}\cdot MOS_{b,2}=3.5555\cdot k_{m,2}$$

(59) $$\phi_{m,1}=k_{m,1}\cdot MOS_{m,1}=4\cdot k_{m,1}$$

(60) $$\phi_{m,2}=k_{m,2}\cdot MOS_{m,2}=5\cdot k_{m,2}$$

Finally, substituting Equations (55) to Equation (60) in Equation (54), we obtain that *G*_1_ ≈ 0.65 and *G*_2_ ≈ 0.85.

**Figure 15:**
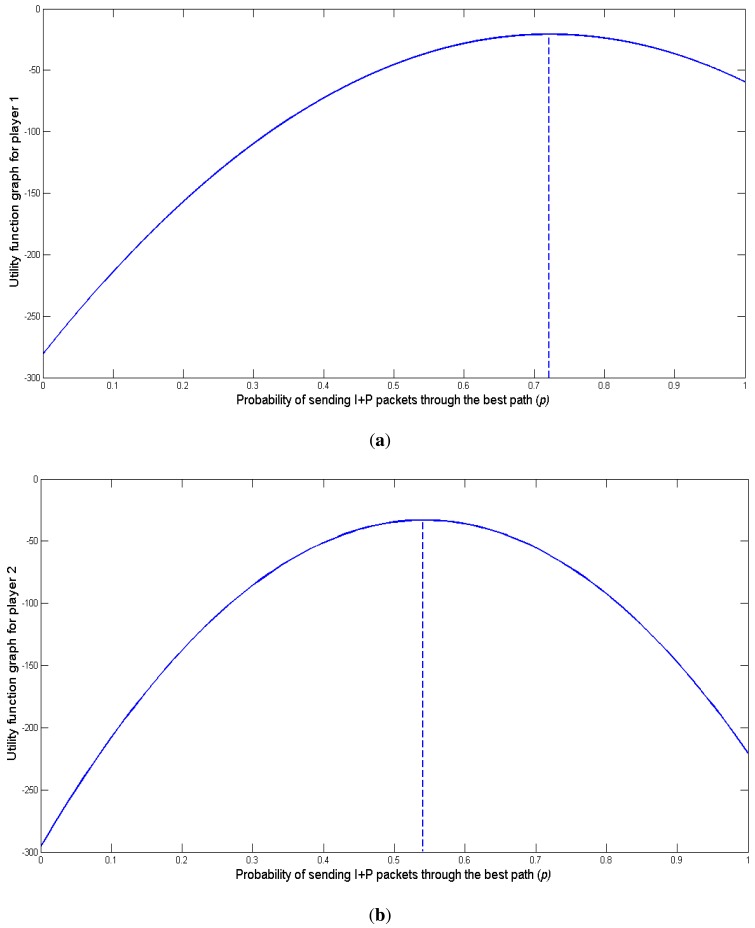
Utility function graph for player 1 and 2. (**a**) Player 1; (**b**) Player 2.

These values mean that for player 1 the gain is 65% more using the game-theoretical model instead of not using it. In the same way, for player 2 the gain is 85%. Figure 15a,b show the utility functions *U*_1_ and *U*_2_ and their maximum values obtained for $p_{i}=p_{i}^{*}$ for players 1 and 2, computed with Equation (26).

#### 7.2.4. Behaviour of $p_{i}^{*}$

It is important to analyze the behavior of $p_{i}^{*}$ when the fraction of packet losses through the best or through the medium-quality paths (*FPL_b_* or *FPL_m_*) changes. We can easily guess that if the packet losses through the best path (*i.e.*, *FPL_b_*) increases, the probability to sent (I+P) frames through that best path $p_{i}^{*}$ should decrease, so more (I+P) packets would be sent through the medium-quality path. On the other hand, if the packet losses through the medium path (*i.e.*, *FPL_m_*) increases, $p_{i}^{*}$ should increase, so more (I+P) packets would be sent through the best path.

**Figure 16:**
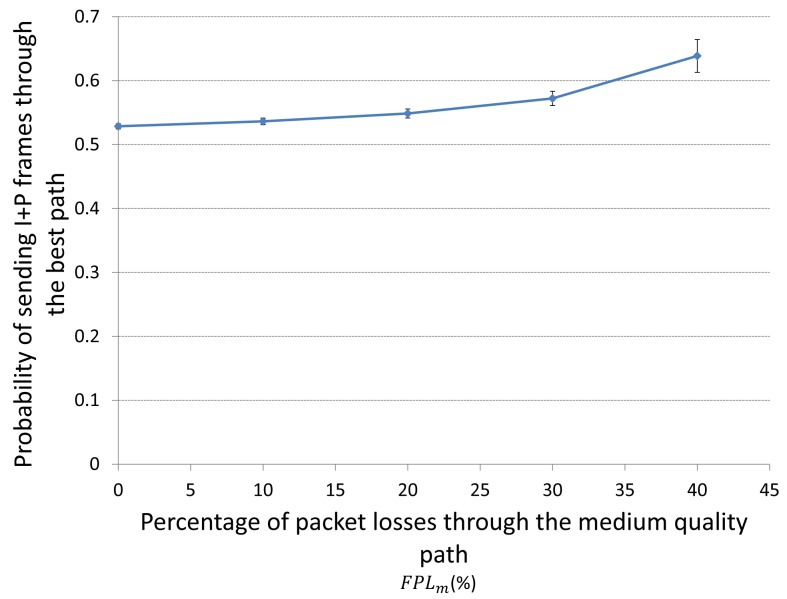
Behaviour of $p_{i}^{*}$ as the fraction of packet losses (*FPL*) through the medium-quality path (*FPL_m_*) increases. Here the FPL through the best path remains constant.

**Figure 17:**
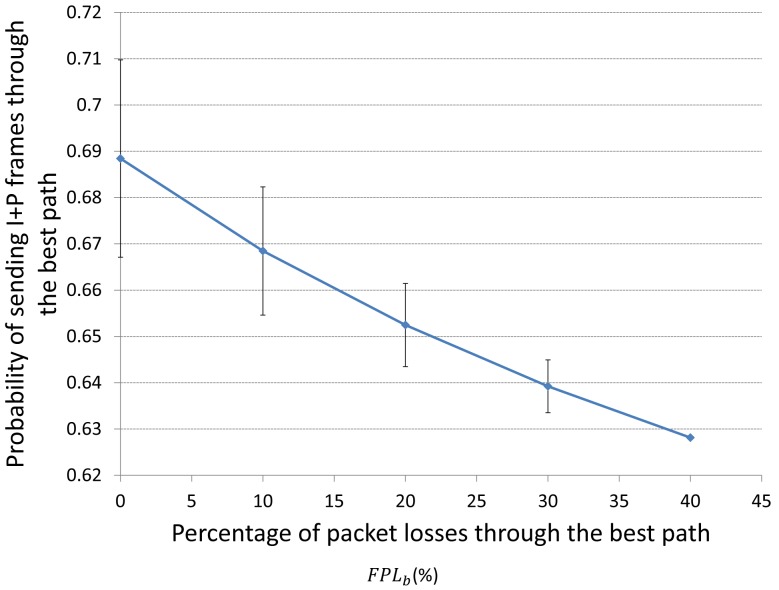
Behaviour of $p_{i}^{*}$ as the fraction of packet losses (*FPL*) through the best path (*FPL_b_*) increases. Here the FPL through the medium-quality path remains constant.

A way to check this logical behavior is to analyze an interval of time of a simulation where jitter and losses behave almost constant in one path (best or medium-quality path) and see how jitter and losses in the other path behave. However, it was impossible to find such an interval. Due to that, we decided to prove it mathematically taking some values of our simulations as inputs. This will help us to analyze the $p_{i}^{*}$ behavior when *FPL_b_* or *FPL_m_* changes, which are shown in Figures 16 and 17. As we can see, we only study the behavior of $p_{i}^{*}$ as a function of the *FPL* till 40% of packet losses since above that threshold it is considered as a non interest range. We show the average results of three different simulations.

Figure 16 depicts the behavior of the best response probability $p_{i}^{*}$ of sending I+P frames through the best path for player *i* when the fraction of packet losses (*FPL*) through the medium-quality path *FPL_m_* increases. It is important to mention that as *FPL_m_* increases, $p_{i}^{*}$ increases as well. The reason is that when *FPL_m_* increases that means that we are loosing more packets in the medium-quality path, so we should better send the packets through the best path (*i.e.*, increase $p_{i}^{*}$) in order to alleviate the congestion in the medium-quality path.

In the same way, Figure 17 depicts the behavior of the best response probability $p_{i}^{*}$ of sending I+P frames through the best path for player *i* (*i.e.*, $p_{i}^{*}$) when the fraction of packet losses (*FPL*) through the best quality path *FPL_b_* increases. We can see that when *FPL_b_* increases, $p_{i}^{*}$ decreases. The reason is that when *FPL_b_* increases that means that we are loosing more packets in the best path, so we should send more packets through the medium-quality pat (*i.e.*, decrease $p_{i}^{*}$) in order to avoid congestion in the best path.

For further information about how we obtained Figures 16 and 17, please see the Appendix B.

In the next section, we present an initial performance evaluation of our proposal in a VANET scenario to show the benefits of our approach in this kind of vehicular scenario. As pointed out in Section 8, we are currently developing a multi-user game-theoretical geographic routing protocol specifically designed to cope with the inherent issues of VANETs, and that will be based on this current work as a starting point.

### 7.3. Performance Evaluation in a VANET Scenario

**Figure 18:**
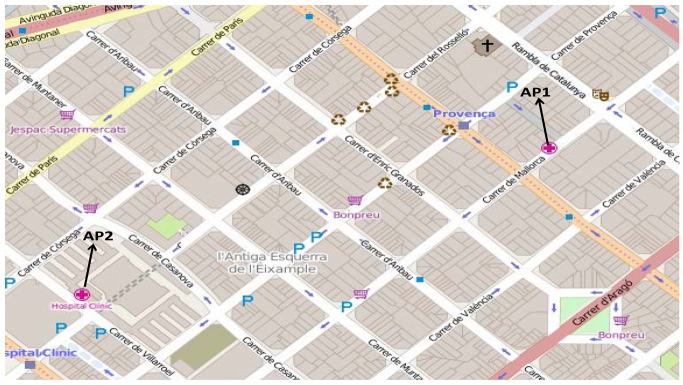
Simulation scenario of Barcelona, Spain (N = 2 users). It includes two emergency units: AP1 (Ana Torres surgery clinic) and AP2 (Hospital Clinic of Barcelona).

**Figure 19:**
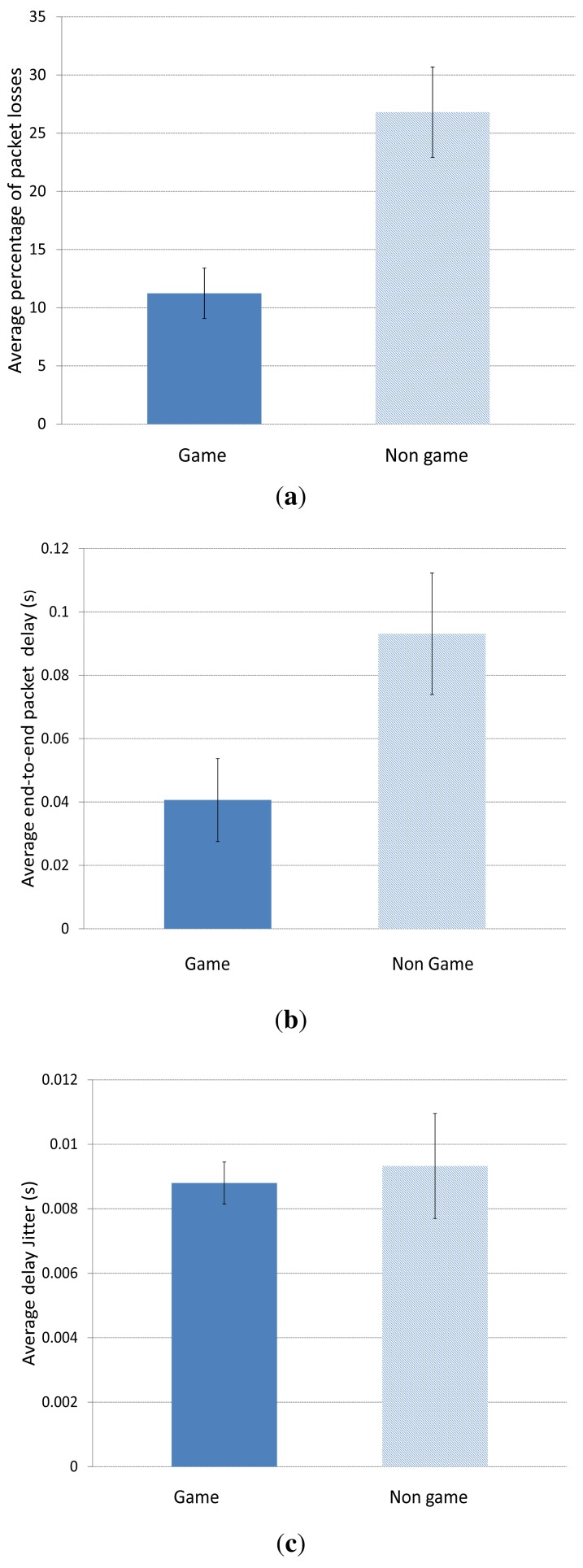
Simulation results for *N* = 2 users. (**a**) Average percentage of packet losses; (**b**) Average end-to-end packet delay; (**c**) Average delay jitter.

In this subsection we introduce a brief performance evaluation of our proposal in a VANET scenario, where we proved the MMDSR protocol plus the multi-user game-theoretical approach described in Section 4. Video flows are transmitted from two vehicles to two access points AP1 and AP2 (see Figure 18), respectively. AP1 is the Ana Torres Institute (a surgery clinic) and AP2 is the Hospital Clinic of Barcelona, which represent two emergency units where vehicles will send their multimedia warning messages upon the event of a traffic accident. Each vehicle will send its multimedia warning message to the closest AP We carried out ten simulations per point using the NS2 [35]. Figure 19 shows the results with confidence intervals (CI) of 90 percent. In the simulations, we used a real city area obtained from the example district of Barcelona, Spain (see Figure 18). In order to simulate a realistic scenario, the CityMob for Roadmaps (C4R) [38] simulator was used to obtain the mobility model. C4R is a mobility generator that uses the Simulation of Urban MObility (SUMO) engine [39]. Besides, C4R imports maps directly from OpenStreetMap [40] and generates NS2 compatible files to specify the mobility model for the vehicles through the city along the whole simulation. The simulation settings of the VANET scenario are shown in Table 6.

Figure 19a shows the average percentage of packet losses for *N* = 2 users when using the game-theoretical scheme against the case of non using it (*Non game* option). We can see how including the game-theoretical routing scheme, the average video packet losses are reduced from 26% to 11%. This decrement of the average packet losses is due to the optimal selection of paths based on a probability value (*i.e.*, p*) that optimally balances the load among the two paths at stake (*i.e.*, the best and the medium-quality paths) depending on the quality of the paths. Figure 19b depicts the average end-to-end packet delay. We can see that the case including the *Game* scheme improves the delay compared to the *Non Game* case. It is reduced from 0.09 s to 0.04 s. Figure 19c depicts the average jitter delay suffered by the packets. Here, we observe that the jitter using the game-theoretical scheme shows slightly better result compared to the *Non Game* case for 2 players.

This has been a first performance evaluation of our game-theoretical routing approach applied in vehicular ad hoc networks. Results show clear benefits after including our game-theoretical approach in a multipath location-based routing protocol over VANETs. Based on these incipient results, in a next work we will further develop a multi-hop geographical routing protocol for VANETs based on a game-theoretical approach to send video warning messages in a smart city following a similar work strategy made in this paper. Our previous experience with VANET routing protocols [41,42] will be a good starting point since in our design we will consider the special inherent features of VANETs. The main difference with this current work will be the design of the game-theoretical routing algorithm based on a hop-by-hop forwarding strategy instead of on an end-to-end forwarding path strategy as we did in this present work. We foresee enhanced results for VANETs after including this modification.

## 8. Conclusions and Future Work

In this paper, we have designed a new rooting protocol for MANETs to send video-warning messages in a smart city. The routing protocol is based on a game-theoretical scheme for *N* users. Our framework could be used in smart cities where prevention of accidents is an important goal. We understand that with a video message, the level of seriousness of the accident could be much better evaluated by the authorities allowing a fast warning of the incident to emergency units, which potentially could save lives.

The users of the framework could be any dynamic sensor such as citizens with smart phones or tablets that could participate in the MANET to send the video to the competent authorities. Also, smart citizens would easily be warned by other citizens about any situation in the city, which would improve the quality of life in the smart cities.

In our framework, the probability *p* of sending the most important video frames (*i.e.*, I+P frames) through the best available path varies depending on some network characteristics. This way, instead of sending the I+P video frames always through the best available path, users play a strategic routing game where these frames will be sent through one of the two best paths according to a certain probability p*.

Simulation results in the MANET scenario show the benefits of our proposal by outperforming the results compared to the case of non using our game-theoretical routing. In terms of packets losses, delay and jitter, results notably improve for *N* = 2, 3, 4 and 5 users, due to the new way of selecting the forwarding path based on p*. Our proposal makes the network more efficient as well as achieves a higher degree of satisfaction of the users by receiving much more (I+P) frames with a lower average end-to-end delay and jitter. This definitively will improve the quality of the video perceived by the end user. Moreover, improvements are also shown for *N* = 2 users in the simulation results of a VANET scenario, in terms of percentage of packet losses, average packet delay and average delay jitter.

As a future work, we are planning a further contribution with a multi-user game-theoretical forwarding algorithm included in the operation of a geographical routing protocol specially designed to distribute multimedia data over VANETs in smart cities. In this kind of scenarios, video-streaming services are taking an important attention from the interest to distribute light and short videos to allow drivers, passengers, paramedics, and first responder teams to capture, share, and watch video sequences from accidents and other emergencies that happened in the city. This way, multimedia warning messages sent upon the event of a traffic accident will allow the emergency units to make a better preliminary evaluation of the situation. Our proposal could be a solution to improve the routing operation to distribute multimedia data over VANETs in smart cities.

In addition, as a future work, the weights of the QoS metrics will be self-configured. We will design a new way to compute and update those weights so that paths/vehicles (paths in MANETs and vehicles in VANETs) would be better arranged according to their QoS metrics and the current state of the environment. In this way, each time the forwarding algorithm needs to classify paths/vehicles, a proper weight value to each metric will be updated depending on the current state of the network.

## Figures and Tables

**Table 1:** Definitions of the variables presented in Equation (14).

**Variable**	**Definition**
*i* = 1, 2, 3…, *N*	is a generic player, being *N* the number of players
*p_i_*	Player's *i* probability of sending the (I+P) frames through his/her best path
*ϕ_b,i_*	Player's *i* benefit for the best path
*ϕ_m,i_*	player's *i* benefit for the medium-quality path
*n_sb,i_*	Number of (I+P) frames sent by player *i* through the best path
*n_rb,i_*	Number of (I+P) frames received from player *i* through the best path
*n_sm,i_*	Number of (I+P) frames sent by player *i* through the medium-quality path
*n_rm,i_*	Number of (I+P) frames received from player *i* through the medium-quality path
*J_b,i_*	Delay jitter of player *i* through the best path
*J_m,i_*	Delay jitter of player *i* through the medium-quality path

**Table 2:** Mapping of mean opinion scores (MOS) and fraction of packet losses (FPL) for (I+P) frames in a general MANET scenario.

**MOS**	**FPL**
5-Excellent	FPL<20%
4-Good	20% ≤ FPL < 30%
3-Fair	30% ≤ FPL < 40%
2-Poor	40% ≤ FPL < 50%
1-Bad	FPL ≥ 50%

**Table 3:** Simulation settings of the MANET scenario.

**Area**	**520** × **520 m^2^**
Number of nodes	50
Average node speed	2 m/s
Transmission range	120 m
Mobility Pattern	Random Waypoint
MAC specification	IEEE 802. 11e,EDCF
*CW_i,min_*, i ∈ (0,1,2,3)	(7,15,31,63)
*CW_i,max_*, i ∈ (0,1,2,3)	(15, 31,1023,1023)
*AIFS_i_*, i ∈ (0,1,2,3)	(34 *μ*s, 43 *μ*s, 52 *μ*s, 61 *μ*s)
(*BW_min_*, *L_max_*, *D_max_*, *J_max_*)	(50 Kbps, 35 %, 0.125 s, 0.004 s)
Nominal bandwidth	11Mbps
Simulation time	1000 s
Video encoding	MPEG-2 VBR
Video bit rate	150 Kbps
Video sources	2 to 5
Video sequence sent	Traffic accidents [37]
Routing protocol	Game-theoretical algorithm + MMDSR
Transport protocol	RTP/RTCP/UDP
Maximum packet size	1500 Bytes
Multipath scheme	*K*=3 paths
Weighting values (Equation (3))	1/7
Queue sizes	50 packets
Interfering CBR traffic	(300, 200, 150 and 120) Kbps
Channel noise	−92 dBm
Mobility pattern generator	Bonnmotion

**Table 4:** Simulation output values for *N* = 2 users.

*J_b_*_,1_, *J_b_*_,2_	(0.021067 s, 0.008867 s)
*J_m_*_,1_,*J_m_*_,2_	(0.011379 s, 0.010136 s)
*n̂_b_*_,1_,*n̂_b_*_,2_	(0.6, 0.45)
*n̂_m_*_,1_,*n̂_m_*_,2_	(0.2, 0.4)
*MOS_b_*_,1_, *MOS_b_*_,2_	(5,5)
*MOS_m_*_,1_,*MOS_m_*_,2_	(4,5)

**Table 5:** Best response probabilities $p_{i}^{*}$ for *N* = 2 users.

*k_b/m_*_,1_, *k_b/m_*_,2_	(0.6268,0.7111)
$p_{1}^{*}$, $p_{2}^{*}$	(0.72, 0.54)

**Table 6:** Simulation settings of the VANET scenario.

**Map Zone**	**Example District of Barcelona**
Area	850 × 580 m^2^
Number of nodes	50
Transmission range	120 m
Mobility generator	SUMO [39]/C4R [38]
MAC specification	IEEE 802.11p
Nominal bandwidth	11 Mbps
Simulation time	250 s
Video encoding	MPEG-2 VBR
Video bit rate	150 Kbps
Video sources	2
Video sequence sent	Traffic accidents [37]
Routing protocol	Game-theoretical algorithm + MMDSR
Transport protocol	RTP/RTCP/UDP
Maximum packet size	1500 Bytes
Multipath scheme	*K* = 3 paths
Weighting values (Equation (3))	1/7
Queue sizes	50 packets
Interfering CBR traffic	100 Kbps

## Appendix

### Appendix A

In this appendix, we describe the test that we made to attain Figure 6. We followed the ITU-T recommendation P.800 (1996) [43] to make our test.

In this test, ten people (7 men and 3 women, aged 25–50) from the department of Telematics Engineering of the Universitat Politècnica de Catalunya (UPC) participated. We asked them to give their mean opinion score (MOS) value to a video received through a MANET urban scenario (see Table 3) when the fraction of packet losses (*FPL*) increased. The video sequence used in the tests was the “Traffic accidents” [37]. Users took into consideration that they were in a MANET scenario, were nodes move, links break often and the network topology is dynamic. The average of the results are shown in Table 2 and Figure 6.

### Appendix B

To compute $p_{i}^{*}$ with Equations (26) and (50) and to attain Figures 16 and 17, we designed a expression to relate the *MOS* with the *FPL*. To do that, we used all the values obtained from the video test depicted in the Appendix A, see Figure 6, and we applied a lineal regression method to derive an equation to approximate the graph shown in Figure 6.

(B1) MOS≃c·FPL+d

where *c* = −8.1081 and *d* = +5.2703.

To draw Figure 16, we made constant all the parameters of Equation (26) except *FPL_m_*. These constant values were taken from simulations. To evaluate the behaviour of $p_{i}^{*}$ when *FPL_m_* increases, we used Equation (B1) to be substituted in Equation (26). Similarly, to draw Figure 17, we made constant all parameters of Equation (26) except *FPL_b_*. Now, to evaluate the behaviour of $p_{i}^{*}$ when *FPL_b_* increases, we used Equation (B1) to be substituted in Equation (26).
